# Rigidity of Stratospheric Travelling Waves

**DOI:** 10.1007/s00220-025-05368-5

**Published:** 2025-07-02

**Authors:** Adrian Constantin, Hua Shao, Hao Zhu

**Affiliations:** 1 Faculty of Mathematics, University of Vienna, Oskar-Morgenstern-Platz 1, 1090 Vienna, Austria; 2 Department of Mathematics, Nanjing University of Aeronautics and Astronautics, Nanjing, 211106 Jiangsu People’s Republic of China; 3 School of Mathematics, Nanjing University, Nanjing, 210093 Jiangsu People’s Republic of China

## Abstract

We present some rigidity results for travelling waves that propagate zonally in bands of latitude in the stratosphere of the outer planets. Such waves arise as perturbations of a background zonally shear flow. The *f*-plane and $\beta$-plane approximations are adequate for the small and moderately wide bands on Jupiter and Saturn, while for the broad zonal jets on Uranus and Neptune one must use spherical coordinates. We show that within the *f*-plane setting the wave speeds must lie in the range of the flow’s zonal velocity, while in the $\beta$-plane setting they cannot exceed the maximum of the zonal velocity but may be slightly less than its minimum. In spherical coordinates we obtain a rigidity result for travelling waves near a zonal flow. By means of some examples, we also show that such zonally travelling waves do not have to be zonally symmetric, and symmetry breaking may occur due to the rotation.

## Introduction

**Fig. 1:**
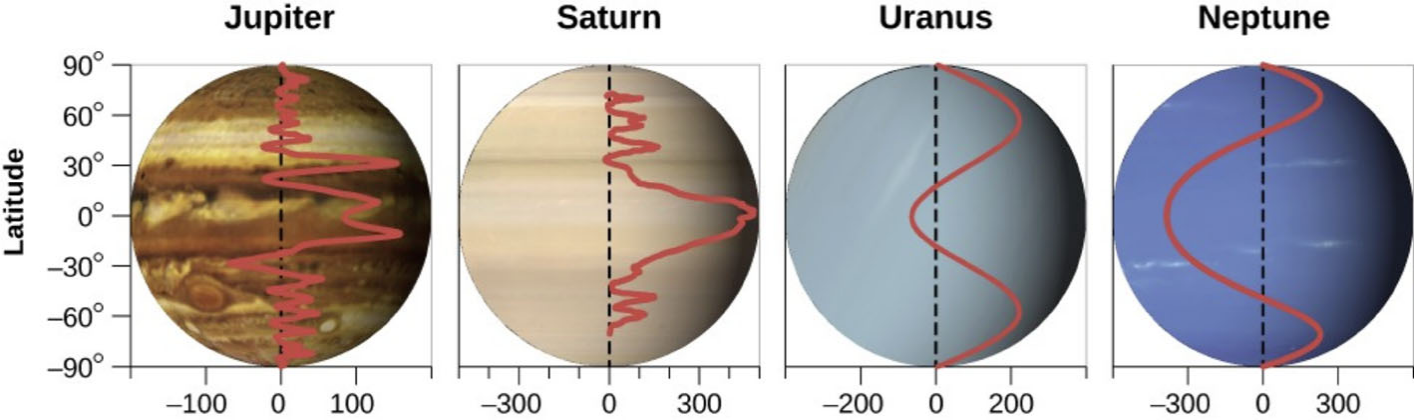
The zonal wind profile of the outer planets, measured in m/s relative to each planet’s rotation speed (Credit: Open-Stax CNX). The most prominent visual features on Jupiter and Saturn are their zonal bandings, called “zones" (with an eastward jet along their poleward boundary and a westward one on the boundary nearest the equator) and “belts" (with the direction of the jets along their boundary reversed); on Jupiter, they can be visualized by their color contrast (bright, respectively dark regions). Uranus and Neptune also display patterns of planetary zonal banding above the tropospheric cloud level, but with a simpler structure—a broad westward equatorial flow and an eastward flow at higher latitudes in each hemisphere

Zonal jets—geophysical steady flows propagating practically in the East-West direction and moving at a speed different from that of its surroundings—are ubiquitous in the planetary flow dynamics of our solar system. They are often the largest, most persistent and hence most recognisable atmospheric features. On the other hand, due to the presence of land masses and to bathymetry variations, the Earth’s ocean currents are typically meandering. Nevertheless, zonal jets are found in the equatorial regions of the Earth’s oceans [12, 42] and the Antarctic Circumpolar Current consists of several high-speed, vertically coherent, sea floor-reaching zonal jets, separated by low-speed currents [1, 42]. With respect to atmospheric flows, the stratosphere is primarily distinguished by its stability, being characterised by strong stratification leading to quasi-horizontal motions. Moreover, stratospheric flow is practically inviscid, with the Euler equation on a rotating sphere capturing the leading-order dynamics (see [14]). Note that the familiar pictures of Jupiter and Saturn (see Fig. 1) show the high altitude clouds just beneath their stratosphere, a fact that is very helpful in visualising the dynamics of the respective stratospheric flows. Throughout this paper we investigate zonally travelling waves in the stratosphere. Time-averages of such flows over intervals from years to decades long represent underlying zonal currents in the form of a steady sheared mean flow but often one can also observe zonal jets without wavy perturbations.

Mercury, Venus and Mars lack a stratosphere, and the Earth has the most unpredictable weather in our solar system due to the sea-land differential heating, this being also the reason why the weather patterns in its Southern Hemisphere are more regular (the ratio of land to ocean being about 1/4, compared to a 1/1.5 northern value). While the zonal flow in the Earth’s northern stratosphere is perturbed by waves propagating up from the troposphere, which break and thus create stratospheric regions with significant meridional velocities [24, 38], during the austral winter and spring, the stratospheric polar vortex in the Earth’s Southern Hemisphere is a zonal jet [36]. As for the outer planets (the gas giants Jupiter and Saturn, and the ice giants Neptune and Uranus), because of their rapid rotation rates and large radii, Coriolis forces dominate convection, so that the meridional flow is much slower than zonal flow [21]. The stratosphere of Jupiter, Saturn and Neptune is dominated by zonal flows that feature a banded structure, with strong zonal jets along their boundaries. Even Uranus, whose rotation axis is tipped 98 degrees relative to its orbit axis, exhibits prominent zonal jets: there is a westward atmospheric flow at low latitudes and an eastward flow at higher latitudes in each hemisphere (only visible in the infrared). With exception of Uranus, whose icy core and sideways position (with the poles where its equator should be) make the temperatures globally uniformly low, impeding the formation of localised flow patterns, immersed within the predominant zonal flow on the outer planets is a plethora of cloud structures, some short-lived but others persisting on large time scales. Examples of symmetric and coherent structures are the Great Red Spot on Jupiter [14], Saturn’s hexagonal circumpolar pattern [7] and the Great Dark Spot on Neptune [18]. Planetary-scale wave disturbances, long-lived and well localised in latitude, were observed on Jupiter, Saturn and Neptune. In contrast to this, data transmitted by the spacecraft Voyager indicates that Uranus appears to have a relatively bland atmosphere—only eight isolated cloud structures were detected, most of them asymmetric and moving as if carried by the zonal mean flow, with no apparent vortex (see [21]), but recent radio telescope observations provide strong evidence for the existence of a vortex anchored at the planet’s north pole (see [2]). Note that rotation controls the weather and dominates solar heating in the visible atmosphere of Uranus—while the planet’s tipped-over position ensures that, averaged over a year, the high latitudes get more sunlight than the low latitudes, the clouds are arrayed in bands parallel to latitude circles and the jets blow in the east–west direction [26].

Jupiter has the most photogenic atmosphere in the solar system, having the most colour contrast of any of the planetary atmospheres, with a cloud-top dynamics easy to track from space. A recent survey of wave-like phenomena on Jupiter [32] shows that the vast majority of observed wave features typically lack symmetry and are found at latitudes with strong prevailing eastward winds, heading in the same direction as Jupiter’s rotation. The typical appearance of waves in regions associated with prograde motions of the mean zonal flow is also noticeable on Saturn and on Neptune. On Jupiter and Saturn, the winds are predominantly eastward (see Fig. 1). The stratospheric waves and vortices on the outer planets can be regarded as perturbations of a background flow consisting of an alternating pattern of eastward and westward zonally shear flows. This background zonal flow appears to be very stable (see the discussion in [10]).

In this paper we prove some rigidity results for zonally stratospheric travelling waves that represent perturbations of an underlying zonal shear flow in a band of latitude. Section 2 is devoted to some preliminary considerations about the governing equations. In Section 3 we investigate the rigidity and symmetry of travelling-wave solutions for the 2D quasi-geostrophic equation (3.1). In the $\beta$-plane setting, adequate for bands of latitude of moderate width (like those delimited by strong zonal jets in the visible atmospheres of Jupiter and Saturn), we prove that the wave speed of a genuine travelling wave cannot exceed the maximum of the zonal velocity (see Theorem 3.2), but can be slightly less than its minimum with a lower bound (see Theorem 3.11). In particular, for any $\beta >0$, $T>0$, and $c\in \mathbb {R}$, we construct non-sheared travelling waves with minimal zonal period *T* and speed *c* less than the minimum of the zonal velocity (see Proposition 3.14). By means of the vorticity-stream function relationships (3.32) and (3.38), we give two further rigidity results in Propositions 3.16 and 3.20. We also construct zonally asymmetric stratospheric travelling waves in Example 3.23 and present a symmetry-breaking phenomenon due to the $\beta$-plane effect in Example 3.24. On the other hand, in zonal bands of a few degrees of latitude it is reasonable to study the dynamics of the atmosphere in the simpler *f*-plane setting. In the *f*-plane setting it turns out that the wave speed of a genuine travelling wave must lie in the range of the zonal velocity (see Proposition 3.3). This fact highlights a qualitative difference in the dynamics of travelling waves between the *f*-plane and the $\beta$-plane settings, indicating that spherical geometry plays an important role, leading to more intricate flows. Indeed, the difference between the two settings is that in the *f*-plane the Coriolis parameter is kept fixed, while the $\beta$-plane captures better the sphericity effect by allowing the Coriolis parameter to vary linearly with the meridional distance. However, the $\beta$-plane approximation is not adequate for the wide zonal jets on Neptune and Uranus, which require the use of spherical coordinates. Very wide zonal jets allow for looser motions and more interactions; for example, Neptune’s Great Dark Spot, whose instantaneous snapshots appear somewhat similar to Jupiter’s Great Red Spot (which is confined to a narrow zonal band—see the discussion in [14]), has a considerable latitudinal drift with time (see [18]) and undergoes dramatic variations in its shape [35]. Nevertheless, some rigidity results near a zonal flow are expected to hold also for the governing equations in spherical coordinates. In Section 4, we consider the Euler equation (4.1) on a rotating sphere and present such a rigidity result for travelling waves near a zonal flow (see Theorem 4.2). We point out the differences of rigidity of travelling waves between the spherical geometry and the flat-space geometry: in contrast to Theorems 3.2 and 3.11 mentioned above, for regions on a sphere rotating with angular speed $\omega$ about the polar axis, the case $\omega =0$ is not distinguished since only the relative position between $-\omega$ and the essential spectra of the linearized operator around the zonal flow is important in Theorem 4.2. Similar to the analysis in the flat-space geometry, we present some rigidity results for flows on a rotating sphere by relying on vorticity-stream function relationships (see Propositions 4.4 and 4.7), and we provide some examples (see Examples 4.8 and 4.9) exhibiting asymmetric wave patterns and the symmetry-breaking phenomenon due to the rotation of the sphere.

Throughout this paper we use the following terminology: shear flows are purely zonal (with zero meridional velocity), while non-sheared travelling waves are oscillating wave patterns that propagate along the east–west direction (eastwards or westwards).

## Preliminaries

In this section, the governing equations for stratospheric flows in spherical coordinates are recalled. Then, for bands of latitude that are either narrow or of moderate width, we describe the governing equations in the *f*-plane and in the $\beta$-plane setting, respectively.

Since the stratospheric flow is inviscid and compressible (see [26]), in a rotating spherical coordinate system $(r',\varphi ,\theta )$, where $\theta \in [-\frac{\pi }{2},\,\frac{\pi }{2}]$ is the angle of latitude, $\varphi \in (-\pi ,\pi ]$ is the angle of longitude, and $r'$ is the distance from the origin at the planet’s center, it is governed by the Euler equation (see [43])

2.1a $$\begin{aligned}&\frac{\mathrm D u'}{\mathrm Dt'} + \frac{u'w'-u'v'\tan \theta }{r'} - 2 \varOmega '(v'\sin \theta -w'\cos \theta ) = -\frac{1}{\rho '}\frac{1}{r'\cos \theta }\frac{\partial p'}{\partial \varphi }\,, \end{aligned}$$

2.1b $$\begin{aligned}&\frac{\mathrm D v'}{\mathrm Dt'} + \frac{v'w'+u'^2\tan \theta }{r'} +2 \varOmega 'u'\sin \theta + \varOmega '^2 r'\sin \theta \cos \theta = -\frac{1}{\rho '}\frac{1}{r'}\frac{\partial p'}{\partial \theta }\,, \end{aligned}$$

2.1c $$\begin{aligned}&\frac{\mathrm D w'}{\mathrm Dt'} - \frac{u'^2+v'^2}{r'} - 2 \varOmega ' u'\cos \theta - \varOmega '^2r'\cos ^2\theta = -\frac{1}{\rho '}\frac{\partial p'}{\partial r'} -g'\,, \end{aligned}$$

coupled with the equation of mass conservation

2.2 $$\begin{aligned} \frac{\mathrm D\rho '}{\mathrm Dt'} + \rho '\left( \frac{1}{r'\cos \theta }\,\frac{\partial u'}{\partial \varphi } + \frac{1}{r'\cos \theta }\,\frac{\partial }{\partial \theta }(v'\cos \theta ) + \frac{1}{r'^2}\,\frac{\partial }{\partial r'}\,(r'^2 w')\right) =0, \end{aligned}$$

the equation of state for an ideal gas

2.3 $$\begin{aligned} p'=\rho '{\mathfrak {R}}'T', \end{aligned}$$

and the first law of thermodynamics

2.4 $$\begin{aligned} c_p' \, \frac{{\mathrm D}T'}{\text {D}t'} -\kappa ' \nabla '^2 T' - \frac{1}{\rho '}\, \frac{{\mathrm D}p'}{{\mathrm D}t'}=Q'. \end{aligned}$$

We use primes to denote physical/dimensional variables; here $(u',v',w')$ are the azimuthal, meridional and vertical components of the velocity field,

$$\begin{aligned} \frac{\mathrm D\phantom {|}}{\mathrm Dt'} = \frac{\partial }{\partial t'}+ \frac{u'}{r'\cos \theta }\frac{\partial }{\partial \varphi } + \frac{v'}{r'}\frac{\partial }{\partial \theta } + w'\frac{\partial }{\partial r'}\, \end{aligned}$$

is the material derivative in spherical coordinates, $p'$ and $\rho '$ are the pressure and density, $\varOmega '$ is the constant rate of rotation of the planet, $g'$ is the (constant) acceleration due to gravity, $T'$ is the (absolute) temperature, ${{\mathfrak {R}}}'$ is the gas constant,

$$\begin{aligned} \nabla '^2 \equiv \frac{\partial ^2}{\partial r'^2} + \frac{2}{r'}\,\frac{\partial }{\partial r'}+ \frac{1}{r'^2}\Big [ \frac{1}{\cos ^2\theta }\,\frac{\partial ^2}{\partial \varphi ^2} + \frac{\partial ^2}{\partial \theta ^2} -\tan \theta \frac{\partial }{\partial \theta }\Big ], \end{aligned}$$

$c_p'$ is the specific heat, $\kappa '/c_p'$ is the thermal diffusivity, while $Q'$ is a general heat-source term.

By means of a dimensional length scale $L'$ for horizontal motion (typically in the range from one degree of latitude to the radius $R'$ of the planet), of typical magnitudes $U'$ and $W'$ for the horizontal and vertical velocities, with $H'$ the mean height and $\overline{\rho }'$ the average density of the stratosphere, we introduce three important flow-parameters:

- the inverse Rossby number $\omega =\frac{\varOmega 'L'}{U'}$;
- the shallowness parameter $\mu =\frac{H'}{L'}$;
- the ratio $\delta =\frac{W'}{U'}$ between the vertical and horizontal velocity scales.

The physically relevant regime for the thin-shell stratosphere is

2.5 $$\begin{aligned} \delta \ll \mu \ll 1. \end{aligned}$$

In particular, the vertical velocity is very small in the stratosphere (typically $\delta$ being of the order of $10^{-5}$), so that a suitable time scale is $L'/U'$, determined by the horizontal flow. Defining dimensionless variables *t*, *z*, *u*, *v*, *w*, $\rho$ and *p* by

2.6 $$\begin{aligned} t' =\displaystyle \frac{L'}{U'}\,t,\ r' =R'+H'z,\ (u',v') =U'(u,v),\ w' =W'w,\ \rho ' = \overline{\rho '}\rho , \ p'=\overline{\rho '}U'^2 p,\nonumber \\ \end{aligned}$$

we obtain from () to (2.2) the components of the non-dimensional Euler equation

2.7 $$\begin{aligned}&\displaystyle \frac{\mathrm D u}{\mathrm Dt} + \frac{\delta uw-uv\tan \theta }{1+\mu z} - 2 \omega (v\sin \theta - \delta w\cos \theta ) = -\frac{1}{\rho }\frac{1}{(1+\mu z)\cos \theta }\, \frac{\partial p}{\partial \varphi }, \end{aligned}$$

2.8 $$\begin{aligned}&\displaystyle \frac{\mathrm D v}{\mathrm Dt} + \frac{\delta vw+u^2\tan \theta }{1+\mu z} +2 \omega u \sin \theta + \omega ^2 (1+\mu z) \sin \theta \cos \theta = -\frac{1}{\rho }\frac{1}{1+\mu z} \, \frac{\partial p}{\partial \theta }, \end{aligned}$$

2.9 $$\begin{aligned}&\displaystyle \mu \delta \, \frac{\mathrm D w}{\mathrm Dt} - \mu \,\frac{u^2+v^2}{1+\mu z} - 2 \mu \omega u \cos \theta - \mu \omega ^2 (1+\mu z)^2 \cos ^2\theta = -\frac{1}{\rho }\frac{\partial p}{\partial z} -g \,, \end{aligned}$$

and the non-dimensional equation of mass conservation

2.10 $$\begin{aligned} \frac{\mathrm D\rho }{\mathrm Dt} + \rho \Big \{\frac{1}{(1+\mu z)\cos \theta }\,\frac{\partial u}{\partial \varphi }&+ \frac{1}{(1+\mu z)\cos \theta }\,\frac{\partial }{\partial \theta }(v\cos \theta ) \nonumber \\&+ \frac{\delta }{\mu }\frac{1}{(1+\mu z)^2}\,\frac{\partial }{\partial z}\,\Big ((1+\mu z)^2 w\Big )\Big \}=0\,, \end{aligned}$$

where

$$\begin{aligned} \frac{\mathrm D\phantom {|}}{\mathrm Dt} = \frac{\partial }{\partial t}+ \frac{u}{(1+\mu z) \cos \theta }\frac{\partial }{\partial \varphi } + \frac{v}{1+\mu z}\frac{\partial }{\partial \theta } + \frac{\delta }{\mu }\,w\frac{\partial }{\partial z} \quad \text {and}\quad g=\frac{g' H'}{U'^2}. \end{aligned}$$

The non-dimensional versions of the governing equations (2.3) and (2.4) are not needed. Indeed, for any solution $(u,v,w,\rho ,p)$ of the system (2.7)–(2.10), (2.6) provides the physical variables $(u',v',w',\rho ',p')$ and then the ideal gas law (2.3) specifies the corresponding temperature, while the second law of thermodynamics (2.4) identifies the associated heat source.

Due to (2.5), at leading-order, the flow dynamics in the thin-shell stratosphere is governed by the limit $\mu \rightarrow 0$ of the non-dimensional equations (2.7)-(2.10):

$$\begin{aligned}&\frac{\partial u_0}{\partial t}+ \frac{u_0}{\cos \theta }\frac{\partial u_0}{\partial \varphi }+ v_0\frac{\partial u_0}{\partial \theta } - u_0v_0\tan \theta - 2\omega \, v_0\sin \theta = -\frac{1}{\rho _0 \cos \theta }\frac{\partial p_0}{\partial \varphi }\,,\\&\frac{\partial v_0}{\partial t}+\frac{u_0}{\cos \theta }\frac{\partial v_0}{\partial \varphi }+ v_0\frac{\partial v_0}{\partial \theta } + u_0^2\tan \theta + 2\omega \, u_0\sin \theta + \omega ^2 \sin \theta \cos \theta = - \frac{1}{\rho _0}\frac{\partial p_0}{\partial \theta }\,, \\&0 = \frac{1}{\rho _0}\frac{\partial p_0}{\partial z} +g \,,\\&\displaystyle \frac{\partial \rho _0}{\partial t}+ \frac{u_0}{\cos \theta }\frac{\partial \rho _0}{\partial \varphi }+ v_0\frac{\partial \rho _0}{\partial \theta } + \frac{\rho _0}{\cos \theta } \Big (\frac{\partial u_0}{\partial \varphi } + \frac{\partial }{\partial \theta }(v_0\cos \theta ) \Big )=0\,, \end{aligned}$$

which simplify to

2.11a $$\begin{aligned}&\frac{\partial u_0}{\partial t}+ \frac{u_0}{\cos \theta }\frac{\partial u_0}{\partial \varphi }+ v_0\frac{\partial u_0}{\partial \theta } - u_0v_0\tan \theta - 2\omega \, v_0\sin \theta = -\frac{1}{\rho _0 \cos \theta }\frac{\partial p_0}{\partial \varphi }\,, \end{aligned}$$

2.11b $$\begin{aligned}&\frac{\partial v_0}{\partial t}+\frac{u_0}{\cos \theta }\frac{\partial v_0}{\partial \varphi }+ v_0\frac{\partial v_0}{\partial \theta } + u_0^2\tan \theta + 2\omega \, u_0\sin \theta + \omega ^2 \sin \theta \cos \theta = - \frac{1}{\rho _0}\frac{\partial p_0}{\partial \theta }\,, \end{aligned}$$

2.11c $$\begin{aligned}&0 = \frac{1}{\rho _0}\frac{\partial p_0}{\partial z} +g \,,\end{aligned}$$

2.11d $$\begin{aligned}&\frac{\partial u_0}{\partial \varphi } + \frac{\partial }{\partial \theta }(v_0\cos \theta ) =0\,, \end{aligned}$$

since

$$\begin{aligned} \rho _0=\rho _0(z) \end{aligned}$$

because stratospheric density changes are practically confined to the vertical direction.

At every fixed height *z*, the system () describes inviscid flow on the surface of a rotating sphere. The relevance of Euler equation on a rotating sphere for planetary-scale stratospheric flows (that is, with the choice $L'=R'$) was recently established in [10]. The above considerations extend the validity of this dynamical feature to the regime $L' \gg H'$, thus facilitating the analysis of large-scale stratospheric flows confined to zonal bands. For example, if the band is of limited latitudinal extent, then the effects of sphericity can be neglected and one can use a locally Cartesian representation of the system (). For this, we define a plane tangent to the spherical surface at a reference point of longitude $\varphi _0$ and latitude $\theta =\theta _0$. This permits us to introduce a Cartesian coordinate system $(x',y')$ in dimensional variables, pointing in the east–west and south-north directions, respectively (see [43]), with

$$\begin{aligned} x'=R'(\varphi -\varphi _0)\,\cos \theta _0=L'x,\qquad y'=R'(\theta -\theta _0)=L'y, \end{aligned}$$

defining the non-dimensional Cartesian coordinates

$$\begin{aligned} x=\frac{R'\cos \theta _0}{L'}\,(\varphi -\varphi _0),\quad y=\frac{R'}{L'}\,(\theta -\theta _0). \end{aligned}$$

The motion on this (*x*, *y*)-tangent plane, with $U_0$ and $V_0$ the azimuthal and meridional velocity components, is governed by the *f*-plane approximation

2.12a $$\begin{aligned}&\frac{\partial U_0}{\partial t}+ U_0\,\frac{\partial U_0}{\partial x}+ V_0\,\frac{\partial U_0}{\partial y} - f_0\, V_0 = -\frac{1}{\rho _0}\,\frac{\partial P_0}{\partial x}\,,\end{aligned}$$

2.12b $$\begin{aligned}&\frac{\partial V_0}{\partial t}+ U_0\,\frac{\partial V_0}{\partial x}+ V_0\,\frac{\partial V_0}{\partial y} + f_0\, U_0 = -\frac{1}{\rho _0}\,\frac{\partial P_0}{\partial y}\,,\end{aligned}$$

2.12c $$\begin{aligned}&0 = \frac{\partial P_0}{\partial z} +g \,,\end{aligned}$$

2.12d $$\begin{aligned}&\frac{\partial U_0}{\partial x} + \frac{\partial V_0}{\partial y} =0\,, \end{aligned}$$

where $f_0=2\omega \,\sin \theta _0$ is the (constant) Coriolis parameter of the flow region of limited latitudinal extent. For localized regions of somewhat larger latitudinal extent around a reference latitude $\theta _0$, to capture the dynamical consequences of the variation of the vertical component of the Coriolis force, one replaces the constant Coriolis parameter $f_0$ in () by

2.13 $$\begin{aligned} f_0 + \beta y \quad \text {with}\quad \beta = \frac{2\Omega '(L')^2\,\cos \theta _0}{U'R'}. \end{aligned}$$

This leads to the $\beta$-plane approximation

2.14a $$\begin{aligned}&\frac{\partial u}{\partial t}+ u\,\frac{\partial u}{\partial x}+ v\,\frac{\partial u}{\partial y} - (f_0 + \beta y)\, v = -\frac{1}{\rho _0}\,\frac{\partial P_0}{\partial x}\,, \end{aligned}$$

2.14b $$\begin{aligned}&\frac{\partial v}{\partial t}+ u\,\frac{\partial v}{\partial x}+ v\,\frac{\partial v}{\partial y} + (f_0 + \beta y)\, u = -\frac{1}{\rho _0}\,\frac{\partial P_0}{\partial y}\,, \end{aligned}$$

2.14c $$\begin{aligned}&0 = \frac{\partial P_0}{\partial z} +g\,, \end{aligned}$$

2.14d $$\begin{aligned}&\frac{\partial u}{\partial x} + \frac{\partial v}{\partial y} =0\,, \end{aligned}$$

where *u* and *v* are the velocity components along the *x* (zonal) and *y* (meridional) coordinates. The system (), conveying that sphericity combined with rotation is dynamically equivalent to a differentially rotating system, captures the most important dynamical effects of sphericity, without the complicating geometric effects, which can be ignored at leading order (see [43]).

Note that (2.13) is obtained from the truncated Taylor expansion

$$\begin{aligned} 2\omega \, \sin \theta = 2\omega \, \sin \big (\theta _0 + (\theta -\theta _0)\big ) \approx 2\omega \,\sin \theta _0 + 2\omega \,(\theta -\theta _0)\,\cos \theta _0 \end{aligned}$$

by recalling the definition of $\omega$ and invoking the tangent-plane approximation

2.15 $$\begin{aligned} \theta -\theta _0 = \frac{yL'}{R'} \end{aligned}$$

for $\theta$ near $\theta _0$. This shows that generally $\beta \ge 0$, with the case $\beta =0$ corresponding to the *f*-plane approximation. With $\Omega ' \approx 1.6 \times 10^{-4}$ rad/s, $U' \approx 150$ m/s and $R' \approx 6 \times 10^7$ m, by choosing $L'$ of the form $\alpha R'$ with $\alpha \in \big [\frac{\pi }{180},\,1 \big ]$, we see from (2.13) that for the zonal bands of Jupiter and Saturn all numerical values $\beta \in [0,120]$ are physically relevant. On the other hand, the $\beta$-plane approximation is not adequate to investigate the broad zonal flows on Uranus and Neptune – for these ones we have to rely on the system () in spherical coordinates.

In each of the systems (), () and () we may regard *z* as a parameter, restricting ourselves to the study of a purely spherical or horizontal motion (for a fixed value of *z*), the change with height being encoded in the hydrostatic dependence of the pressure on the *z*-variable. To simplify the notation, from now on we suppress the dependence on the *z*-variable.

## 2D Quasi-Geostrophic Flows

Within the $\beta$-plane and the *f*-plane settings, we investigate the rigidity of stratospheric travelling waves in Subsection 3.1, and study the zonal asymmetry of travelling waves and the symmetry-breaking phenomenon in Subsection 3.2. More precisely, in Subsection 3.1 we prove that the propagation speed of a travelling wave must lie in the range of its zonal velocity in the *f*-plane setting, while the speed cannot exceed the maximum of the zonal velocity but can be slightly less than its minimum in the $\beta$-plane setting. This difference is emphasized by constructing in the $\beta$-plane setting genuine travelling waves with speeds less than the minimum of the zonal velocity. Moreover, we establish vorticity-stream function relationships for a class of travelling waves and prove some rigidity results that rely on them. In Subsection 3.2, we construct zonally asymmetric travelling waves and exhibit the symmetry-breaking phenomenon due to the $\beta$-plane effect.

We write the incompressible 2D-system (2.14a)-(2.14b) as

3.1 $$\begin{aligned} \partial _{t}\vec {u}+(\vec {u}\cdot \nabla )\vec {u}=-\nabla P-\beta yJ\textbf{u},\quad \nabla \cdot \vec {u}=0, \end{aligned}$$

where $\beta \ge 0$, $\vec {u}(x,y)=(u(x,y),v(x,y))$ is the fluid velocity, $P=\frac{P_0}{\rho _0}-f_0\psi$ is the modified pressure and $$ J= \begin{pmatrix} 0 &  -1\\ 1 &  0 \end{pmatrix} $$ is the rotation matrix, since the incompressibility condition (2.14d) ensures the existence of a stream function $\psi$ with

3.2 $$\begin{aligned} (u,v)=(-\psi _y,\psi _x)\,. \end{aligned}$$

We study the fluid flow in the domain

$$\begin{aligned} \Omega =\big \{(x,y): x\in \mathbb {T}_{q\pi }={\mathbb {R}}/{q\pi {\mathbb {Z}}},\ y\in {[-d,d]}\big \} \end{aligned}$$

with the non-permeable boundary condition:

3.3 $$\begin{aligned} v=0\quad \text {on}\quad y=\pm d, \end{aligned}$$

where $q\in (0,2]$ and $d>0$. In view of (2.15), this setting describes stratospheric flows in zonal bands. The vorticity form of (3.1), which is also called the 2-dimensional quasi-geostrophic potential vorticity equation, has the form

3.4 $$\begin{aligned} \partial _{t}\gamma +(\vec {u}\cdot \nabla )\gamma +\beta v =0, \end{aligned}$$

where $\gamma =\partial _{x}v-\partial _{y}u=\Delta \psi$ is the vorticity. Denote

$$\begin{aligned} F_{\max }=\max _{x\in \mathbb {T}_{q\pi },y\in [-d,d]}\{F(x,y)\},\quad F_{\min }=\min _{x\in \mathbb {T}_{q\pi },y\in [-d,d]}\{F(x,y)\}\, \end{aligned}$$

for a function $$F \in C(\mathbb {T}_{q\pi }\times [-d,d])$$.

### Rigidity of travelling wave solutions for 2D quasi-geostrophic equation

The recent survey [32] for the visible atmosphere of Jupiter seems to indicate the absence of prograde wave-disturbances propagating zonally at speeds that exceed those of the underlying background flow. This feature holds for some explicit non-trivial solutions to (3.4).

#### Example 3.1

Consider the region $\mathbb {T}_{2\pi }\times [-1,1]$. One can easily check that if $\psi (x,y)$ solves the boundary-value problem

3.5 $$\begin{aligned} {\left\{ \begin{array}{ll} \partial _x^2 \psi + \partial _y^2 \psi + \beta y = G(\psi + cy) ,\\ \partial _x\psi =0 \quad \text {on}\quad y= \pm 1, \end{array}\right. } \end{aligned}$$

for some differentiable function *G*, then $\psi (x-ct,y)$ is a travelling wave solution to (3.4) propagating zonally with speed *c*. Explicit solutions of (3.5) can be found for linear functions $G(\Psi )=\lambda \Psi + \xi$. Indeed, in this case $\partial _x\psi$, a $2\pi$-periodic function in the *x*-variable, solves the classical eigenvalue problem

$$\begin{aligned} {\left\{ \begin{array}{ll} ( \partial _x^2 + \partial _y^2) \partial _x \psi = \lambda \partial _x\psi ,\\ \partial _x\psi =0 \quad \text {on}\quad y= \pm 1. \end{array}\right. } \end{aligned}$$

For integers $n,\, k \ge 1$, a real constant *A*, and

3.6 $$\begin{aligned} \lambda =- n^2 - \frac{(2k+1)^2\pi ^2}{4}, \end{aligned}$$

we find the solutions

$$\begin{aligned} \partial _x \psi (x,y)=A \, \cos \Big ( \frac{(2k+1)\pi y}{2} \Big )\,\cos ( nx). \end{aligned}$$

Consequently, setting $a=\frac{A}{ n}$, we have

3.7 $$\begin{aligned} \psi (x,y)= a \, \cos \Big ( \frac{(2k+1)\pi y}{2} \Big )\,\sin ( nx)+ \psi _0(y), \end{aligned}$$

with the function $\psi _0$ satisfying the ordinary differential equation

$$\begin{aligned} {d^2\over dy^2} \psi _0 = \lambda \psi _0 + (\lambda c -\beta )y + \xi . \end{aligned}$$

Recalling (3.6) and setting $\mu = \sqrt{n^2+\frac{(2k+1)^2}{4}\pi ^2}=\sqrt{-\lambda }$, the variation of constants formula yields

3.8 $$\begin{aligned} \psi _0(y)=\tilde{A} \,\cos (\mu y) + B\,\sin (\mu y) + \frac{\lambda c - \beta }{\mu ^2}\,y+ \frac{\xi }{\mu ^2} \end{aligned}$$

for some real constants $\tilde{A}$ and *B*. From (3.7) to (3.8) and (3.2) we obtain

$$\begin{aligned} \frac{1}{2\pi } \int _{0}^{2\pi } u(x,y)\,dx&= \mu \tilde{A} \sin (\mu y)-\mu B\cos (\mu y)+ \frac{\beta -c\lambda }{\mu ^2}\\&=\mu \sqrt{\tilde{A}^2+B^2}\sin (\mu y-\theta )+\frac{\beta }{\mu ^2} + c \end{aligned}$$

for some $\theta \in \left( -{\pi },{\pi }\right]$. Then

$$\begin{aligned} \max _{y\in [-1,1]}\left( \frac{1}{2\pi } \int _{0}^{2\pi } u(x,y)\,dx\right) \ge \frac{\beta }{\mu ^2} + c\ge c \end{aligned}$$

and

3.9 $$\begin{aligned} u_{\max } \ge c. \end{aligned}$$

Our first result shows that (3.9) must hold for all zonally non-sheared travelling wave solutions of (3.1).

#### Theorem 3.2

A travelling wave solution

$$\begin{aligned} \vec {u}(x-ct,y)=(u(x-ct,y),v(x-ct,y))\in C^2(\mathbb {T}_{q\pi }\times [-d,d]) \end{aligned}$$

of the quasi-geostrophic equation (3.1) with $c>u_{\max }$ can only be a shear flow.

We give two proofs of Theorem 3.2.

#### First proof of Theorem 3.2

Suppose that $c>u_{\max }$. Since the vorticity

$$\begin{aligned} \gamma (x-ct,y)=\partial _{x}v(x-ct,y)-\partial _{y}u(x-ct,y) \end{aligned}$$

solves (3.4), we have

3.10 $$\begin{aligned} (u-c)\partial _{x}\gamma +v\partial _{y}\gamma +\beta v=0. \end{aligned}$$

By the incompressible condition $\nabla \cdot \vec {u}=0$, we have

$$\begin{aligned} \partial _{x}\gamma =\Delta v,\;\partial _{y}\gamma =-\Delta u. \end{aligned}$$

Thus, (3.10) becomes

3.11 $$\begin{aligned} (u-c)\Delta v+(\beta -\Delta u)v=0. \end{aligned}$$

We define

$$\begin{aligned} F=\frac{v}{u-c}. \end{aligned}$$

Then

$$\begin{aligned} \partial _{y}F=\frac{\partial _{y}v(u-c)-v\partial _{y}u}{(u-c)^2}, \end{aligned}$$

which implies

3.12 $$\begin{aligned} \partial _{y}((u-c)^2\partial _{y}F)=\partial _{y}(\partial _{y}v(u-c)-v\partial _{y}u)=\partial _{y}^{2}v(u-c)-v\partial _{y}^{2}u. \end{aligned}$$

Similarly,

$$\begin{aligned}\partial _{x}F=\frac{\partial _{x}v(u-c)-v\partial _{x}u}{(u-c)^2},\end{aligned}$$

which yields

3.13 $$\begin{aligned} \partial _{x}((u-c)^2\partial _{x}F)=\partial _{x}(\partial _{x}v(u-c)-v\partial _{x}u)=\partial _{x}^{2}v(u-c)-v\partial _{x}^{2}u. \end{aligned}$$

Combining (3.12) and (3.13) we get

$$\begin{aligned} \partial _{x}((u-c)^2\partial _{x}F)+\partial _{y}((u-c)^2\partial _{y}F)=(u-c)\Delta v-v\Delta u. \end{aligned}$$

Now, by (3.11) and the definition of *F*, we have

3.14 $$\begin{aligned} \partial _{x}((u-c)^2\partial _{x}F)+\partial _{y}((u-c)^2\partial _{y}F) =-\beta v=-\beta (u-c)F. \end{aligned}$$

Multiplying (3.14) by *F* and integrating over $\mathbb {T}_{q\pi }\times [-d,d]$, we have

3.15 $$\begin{aligned}  &   \int _{-d}^{d}\int _{0}^{q\pi }(\partial _{x}((u-c)^2\partial _{x}F)+\partial _{y}((u-c)^2\partial _{y}F))Fdxdy \nonumber \\  &   \quad =\int _{-d}^{d}\int _{0}^{q\pi }-\beta (u-c)|F|^2dxdy. \end{aligned}$$

The periodic boundary condition in *x* and the boundary condition (3.3) in *y* of *v* yield

3.16 $$\begin{aligned} \quad \text {Left hand side of (3.15)}=&\int _{-d}^{d}\left( ((u-c)^2 F\partial _{x}F)\bigg |_{x=0}^{q\pi }-\int _{0}^{q\pi }|\partial _{x}F|^2(u-c)^2dx\right) dy \nonumber \\&+\int _{0}^{q\pi }\left( ((u-c)^2F\partial _{y}F)\bigg |_{y=-d}^{d}-\int _{-d}^{d}|\partial _{y}F|^2(u-c)^2dy\right) dx \nonumber \\ =&-\int _{-d}^{d}\int _{0}^{q\pi }\left( |\partial _{x}F|^2(u-c)^2+|\partial _{y}F|^2(u-c)^2\right) dxdy\le 0. \end{aligned}$$

On the other hand, by $\beta \ge 0$ and the assumption $c>u_{\max }$, we have

3.17 $$\begin{aligned} \begin{aligned} \text {Right hand side of (3.15)}=\int _{-d}^{d}\int _{0}^{q\pi }-\beta (u-c)|F|^2dxdy\ge 0. \end{aligned} \end{aligned}$$

It follows from (3.16) and (3.17) that

3.18 $$\begin{aligned}  &   \int _{-d}^{d}\int _{0}^{q\pi }\left( |\partial _{x}F|^2(u-c)^2+|\partial _{y}F|^2(u-c)^2\right) dxdy\nonumber \\  &   \quad =\int _{-d}^{d}\int _{0}^{q\pi } \beta (u-c)|F|^2dxdy=0. \end{aligned}$$

Thus $F=0$, which implies $v=0$, so that the travelling wave solution $\vec {u}(x-ct,y)$ has the form $(u(x-ct,y),0)$. The incompressible condition $\nabla \cdot \vec {u}=0$ yields now $\partial _x u=0$ and therefore $\vec {u}(x-ct,y)$ is a shear flow. $\square$

#### Alternative proof of Theorem 3.2

By (3.11), we have

$$\begin{aligned} \int _{-d}^{d}\int _{0}^{q\pi }\left( |\nabla v|^2-{\beta -\Delta u\over u-c}v^2\right) dxdy=0. \end{aligned}$$

Then

3.19 $$\begin{aligned} \int _{-d}^{d}\int _{0}^{q\pi }\left( \left( | v_y|^2+{u_{yy}\over u-c}v^2\right) +\left( | v_x|^2+{u_{xx}\over u-c}v^2\right) -{\beta \over u-c}v^2\right) dxdy=0. \end{aligned}$$

Integration by parts gives

3.20 $$\begin{aligned} \int _{-d}^{d}\int _{0}^{q\pi }{u_{yy}\over u-c}v^2dxdy=\int _{-d}^{d}\int _{0}^{q\pi }\left( -{2vv_yu_y\over u-c}+{v^2u_y^2\over (u-c)^2}\right) dxdy \end{aligned}$$

and

3.21 $$\begin{aligned} \int _{-d}^{d}\int _{0}^{q\pi }{u_{xx}\over u-c}v^2dxdy=\int _{-d}^{d}\int _{0}^{q\pi }\left( -{2vv_xu_x\over u-c}+{v^2u_x^2\over (u-c)^2}\right) dxdy. \end{aligned}$$

Inserting (3.20)-(3.21) into (3.19), we have

$$\begin{aligned} \int _{-d}^{d}\int _{0}^{q\pi }\left( \left( v_y-{vu_y\over u-c}\right) ^2+\left( v_x-{vu_x\over u-c}\right) ^2-{\beta \over u-c}v^2\right) dxdy=0. \end{aligned}$$

This is exactly (3.18) and we can now proceed as in the first proof. $\square$

If $\beta =0$, by inspection we see that the above considerations apply also if $c < u_{\min }$. Thus, we have the following result for the *f*-plane approximation.

#### Proposition 3.3

Let $\beta =0$. For a travelling wave solution

$$\begin{aligned} \vec {u}(x-ct,y)=(u(x-ct,y),v(x-ct,y))\in C^2(\mathbb {T}_{q\pi }\times [-d,d]) \end{aligned}$$

of the quasi-geostrophic equation (3.1), if $c\notin Ran(u)$, then $\vec {u}(x-ct,y)$ is a shear flow.

Spectacular steady flow patterns are visible in the atmospheres of the outer planets. The most striking is Jupiter’s Great Red Spot, confined to a relatively narrow zonal band so that the *f*-plane approximation is relevant for its dynamics (see [14]). Regarding rigidity results for steady stratospheric flows, let us point out that a steady, planar, incompressible and inviscid fluid flow in a channel must be a shear flow if it does not feature stagnation points (see [22]). Since the quasi-geostrophic equation (3.1) with $\beta =0$ is merely an incompressible planar Euler equation for a modified pressure, we obtain at once the following result.

#### Lemma 3.4

For $\beta =0$, if a steady solution $\psi \in C^3(\mathbb {T}_{q\pi }\times [-d,d])$ of the quasi-geostrophic potential vorticity equation (3.4) satisfies

3.22 $$\begin{aligned} \inf _{(x,y)\in \mathbb {T}_{q\pi }\times [-d,d]}\{|\psi _y(x,y)|^2+|\psi _x(x,y)|^2\}>0, \end{aligned}$$

then it is a shear flow.

#### Remark 3.5

According to Lemma 3.4, Jupiter’s Great Red Spot should exhibit a stagnation point. Indeed, the flow is still at the center of this storm (see [14]).

Using Lemma 3.4 and the following simple observation, Proposition 3.3 can be improved.

#### Lemma 3.6

If $\psi (x-ct,y)\in C^3(\mathbb {T}_{q\pi }\times [-d,d])$ is a travelling wave solution of the quasi-geostrophic potential vorticity equation (3.4), then $\psi +cy$ is a steady solution of this equation.

#### Proof

Since $\psi (x-ct,y)$ is a travelling wave solution of equation (3.4), we have

$$\begin{aligned} (-\psi _y-c)\partial _{x}\gamma +\psi _x\partial _{y}\gamma +\beta \psi _x=0. \end{aligned}$$

This can be written in the form

3.23 $$\begin{aligned} -\partial _y(\psi +cy)\partial _{x}(\gamma +\beta y)+\partial _{x}(\psi +cy)\partial _{y}(\gamma +\beta y)=0, \end{aligned}$$

or, using the Poisson-bracket notation, as $\{\psi +cy,\gamma +\beta y\}=0$. Since $\Delta \psi =\gamma$, we obtain

$$\begin{aligned}\{\psi +cy,\Delta (\psi +cy)+\beta y\}=0,\end{aligned}$$

which shows that $\psi +cy$ is a steady solution of (3.4). $\square$

These considerations lead to the following improvement of Proposition 3.3.

#### Proposition 3.7

Let $\beta =0$. For a travelling wave solution $\psi (x-ct,y)\in C^3(\mathbb {T}_{q\pi }\times [-d,d])$ of the quasi-geostrophic potential vorticity equation (3.4), if

3.24 $$\begin{aligned} \inf _{(x,y)\in \mathbb {T}_{q\pi }\times [-d,d]}\{|\psi _y(x,y)+c|^2+|\psi _x(x,y)|^2\}>0, \end{aligned}$$

then $\psi (x-ct,y)$ is a shear flow.

#### Proof

By Lemma 3.6, $\psi +cy$ is a steady solution of (3.4). Lemma 3.4 yields now that $\psi +cy$ and thus $\psi$ is a shear flow. $\square$

#### Remark 3.8

The rigidity result in Proposition 3.7 does not hold for $c \ne 0$ if one replaces condition (3.24) by condition (3.22). An example is the travelling wave solution describing Saturn’s circumpolar hexagon, in which case the absence of stagnation points, (3.22), can be verified directly from the explicit parametric formulas for the velocity field provided in [7]. Consider the region $\mathbb {T}_{2\pi }\times [-1,1]$. Another example is provided by $\psi (x,y)=-cy + Y(x,y)$ with $c>\pi$ and

3.25 $$\begin{aligned} Y(x,y)=\cos (x)\cos \left( \frac{\pi y}{2}\right) , \end{aligned}$$

an eigenfunction for the eigenvalue $\frac{\pi ^2}{4}+1$ of the boundary-value problem

$$\begin{aligned} {\left\{ \begin{array}{ll} -\Delta Y=\lambda Y\quad \textrm{on}& \quad \mathbb {T}_{2\pi }\times [-1,1], \\ \quad Y=0\quad \quad \;\,\textrm{on}&  \quad y=\pm 1. \end{array}\right. } \end{aligned}$$

Indeed, since

$$\begin{aligned} \{\psi +cy,\gamma \}=\left\{ (-c+c)y+Y,-\left( \frac{\pi ^2}{4}+1\right) Y\right\} =0, \end{aligned}$$

we see that $\psi (x-ct,y)$ is a non-sheared travelling wave solution of (3.4) with $\beta =0$. However,

$$\begin{aligned} -\psi _y(x,y)=c+\frac{\pi }{2}\cos (x)\sin \left( \frac{\pi y}{2}\right) \,\quad \text {and}\quad \psi _x(x,y)=-\sin (x)\cos \left( \frac{\pi y}{2}\right) \end{aligned}$$

yield

$$\begin{aligned} |\psi _y|^2+|\psi _x|^2 =&c^2+c\pi \cos (x)\sin \left( \frac{\pi y}{2}\right) +\frac{\pi ^2}{4}\cos ^{2}(x)\sin ^{2}\left( \frac{\pi y}{2}\right) +\sin ^{2}(x)\cos ^{2}\left( \frac{\pi y}{2}\right) \\ \ge&c^2-c\pi >0\,,\qquad (x,y)\in \mathbb {T}_{2\pi }\times [-1,1]\,, \end{aligned}$$

so that (3.22) is satisfied.

The next example invalidates the extension of Lemma 3.4 and that of Proposition 3.3 to $\beta >0$, showing that the dynamics in the wider bands of latitude where the $\beta$-plane framework applies is more intricate than that in the narrower bands that can be studied within the *f*-plane setting.

#### Example 3.9

Consider the region $\mathbb {T}_{2\pi }\times [-1,1]$. Let

$$\begin{aligned} \psi (x,y)=\alpha y+Y(x,y)=\alpha y+\cos (x)\cos \left( \frac{\pi y}{2}\right) \end{aligned}$$

with *Y* given by (3.25), $\beta >\frac{\pi }{2}(\frac{\pi ^2}{4}+1)$ and

$$\begin{aligned} \alpha =\frac{-\beta }{\frac{\pi ^2}{4}+1}. \end{aligned}$$

The vorticity of this flow is $\gamma =\Delta \psi =-(\frac{\pi ^2}{4}+1)Y$ and

$$\begin{aligned} \{\psi ,\gamma +\beta y\}=\left\{ \alpha y+Y,-\left( \frac{\pi ^2}{4}+1\right) Y+\beta y\right\} =0. \end{aligned}$$

Thus $\psi (x,y)$ is a non-sheared steady solution of (3.4), and the range of its zonal velocity

$$\begin{aligned} u(x,y)=-\psi _y(x,y)=\frac{\beta }{\frac{\pi ^2}{4}+1}+\frac{\pi }{2}\cos (x)\sin \left( \frac{\pi y}{2}\right) \end{aligned}$$

is

$$\begin{aligned} Ran(u) =\left[ \frac{\beta }{\frac{\pi ^2}{4}+1}-\frac{\pi }{2},\frac{\beta }{\frac{\pi ^2}{4}+1}+\frac{\pi }{2}\right] \subset (0,\infty ) \end{aligned}$$

so that (3.22) holds.

#### Remark 3.10

In view of Theorem 3.2 and Proposition 3.3, Example 3.9 shows that for sufficiently wide bands of latitude, it should be possible to spot in the visible atmospheres of the outer planets travelling wave patterns that propagate zonally at speeds $c<u_{\min }$. Actually, in December 2010, a giant storm erupted on Saturn in a band of latitude 10000 km wide, located in the middle of a westward jet centered at 35$^\circ$N latitude, and lasted for half a year. During the storm’s six-month lifetime, its leading edge maintained its shape and moved westward a little faster than the underlying zonal flow (see [26]).

The following result shows that, within the $\beta$-plane setting, the speed of a travelling wave propagating westward cannot be much faster than $u_{\min }$. Note that since the wave is a perturbation of the background zonal flow, at a fixed latitude of relevance, the zonal velocity of the underlying shear flow is the time-averaged zonal velocity of the wave.

#### Theorem 3.11

A travelling wave solution

$$\begin{aligned} \vec {u}(x-ct,y)=(u(x-ct,y),v(x-ct,y))\in C^1(\mathbb {T}_{q\pi }\times [-d,d]) \end{aligned}$$

of the quasi-geostrophic equation (3.1) with

3.26 $$\begin{aligned} c<u_{\min }-\frac{2\beta d^2}{\pi ^2}-\frac{2d^2}{\pi ^2}\sqrt{\beta ^2+{\pi ^2\beta \over d^2}(u_{\max }-u_{\min })} \end{aligned}$$

must be a shear flow.

#### Proof

If (3.26) holds, the same considerations leading to (3.15) and (3.16) yield

3.27 $$\begin{aligned} \int _{-d}^{d}\int _{0}^{q\pi }(u-c)^2|\nabla F|^2dxdy=\int _{-d}^{d}\int _{0}^{q\pi }\beta (u-c)|F|^2dxdy. \end{aligned}$$

Since $c<u_{\min }$, we get

$$\begin{aligned} |u-c|=u-c\ge u_{\min }-c>0. \end{aligned}$$

Thus,

3.28 $$\begin{aligned} \text {Left hand side of (3.27)} \ge (u_{\min }-c)^2\int _{-d}^{d}\int _{0}^{q\pi }|\nabla F|^2dxdy. \end{aligned}$$

Taking into account the boundary condition (3.3) in *y* for *v*, we have the Poincaré inequality

3.29 $$\begin{aligned} \int _{-d}^{d}|F_y|^2dy\ge \frac{\pi ^2}{4d^2}\int _{-d}^{d}|F|^2dy. \end{aligned}$$

It follows from (3.27) to (3.29) that

3.30 $$\begin{aligned} \int _{-d}^{d}\int _{0}^{q\pi }\beta (u-c)|F|^2dxdy\ge \frac{\pi ^2}{4d^2}(u_{\min }-c)^2\int _{-d}^{d}\int _{0}^{q\pi }|F|^2dxdy. \end{aligned}$$

This implies that

$$\begin{aligned} \beta (u_{\max }-c)\int _{-d}^{d}\int _{0}^{q\pi }|F|^2dxdy\ge \frac{\pi ^2}{4d^2}(u_{\min }-c)^2\int _{-d}^{d}\int _{0}^{q\pi }|F|^2dxdy, \end{aligned}$$

and therefore

$$\begin{aligned}\left( \beta (u_{\max }-c)-\frac{\pi ^2}{4d^2}(u_{\min }-c)^2\right) \int _{-d}^{d}\int _{0}^{q\pi }|F|^2dxdy\ge 0.\end{aligned}$$

Since $c<u_{\min }-\frac{2\beta d^2}{\pi ^2}-\frac{2d^2}{\pi ^2}\sqrt{\beta ^2+{\pi ^2\beta \over d^2}(u_{\max }-u_{\min })}$, we infer

$$\begin{aligned}\beta (u_{\max }-c)-\frac{\pi ^2}{4d^2}(u_{\min }-c)^2<0.\end{aligned}$$

Thus,

$$\begin{aligned}\int _{-d}^{d}\int _{0}^{q\pi }|F|^2dxdy=0.\end{aligned}$$

This implies $F=0$, so that $v=0$ and (2.14d) ensures now that $\vec {u}$ is a shear flow. $\square$

Note that the lower bound of the minimal possible speed of a zonally travelling wave provided by the right hand side of (3.26) is not sharp for $\beta >0$. In fact, suppose that there exists a non-sheared travelling wave solution $(\tilde{u}(x-\tilde{c}t,y),\tilde{v}(x-\tilde{c}t,y))\in C^1(\mathbb {T}_{q\pi }\times [-d,d])$ of (3.1) with

$$\begin{aligned} \tilde{c}=\tilde{u}_{\min }-\frac{2\beta d^2}{\pi ^2}-\frac{2d^2}{\pi ^2}\sqrt{\beta ^2+{\pi ^2\beta \over d^2}(\tilde{u}_{\max }-\tilde{u}_{\min })}. \end{aligned}$$

Then $\beta (\tilde{u}_{\max }-\tilde{c})=\frac{\pi ^2}{4d^2}(\tilde{u}_{\min }-\tilde{c})^2.$ Similar to (3.30), we have

3.31 $$\begin{aligned} \int _{-d}^{d}\int _{0}^{q\pi }(\tilde{u}-\tilde{c})|\tilde{F}|^2dxdy\ge (\tilde{u}_{\max }-\tilde{c})\int _{-d}^{d}\int _{0}^{q\pi }|\tilde{F}|^2dxdy, \end{aligned}$$

where $\tilde{F}=\frac{\tilde{v}}{\tilde{u}-\tilde{c}}$. Dividing the domain $\mathbb {T}_{q\pi }\times [-d,d]$ into $\{\tilde{u}=\tilde{u}_{\max }\}$ and $\{\tilde{u}<\tilde{u}_{\max }\}$, by (3.31) we have

$$\begin{aligned} \iint _{\{\tilde{u}<\tilde{u}_{\max }\}}(\tilde{u}-\tilde{c})|\tilde{F}|^2dxdy\ge (\tilde{u}_{\max }-\tilde{c})\iint _{\{\tilde{u}<\tilde{u}_{\max }\}}|\tilde{F}|^2dxdy. \end{aligned}$$

This implies $\tilde{F}=0$ and $\tilde{v}=0$ on $\{\tilde{u}<\tilde{u}_{\max }\}$. By the incompressible condition, we know that $\tilde{v}$ is independent of *y* on $\{\tilde{u}=\tilde{u}_{\max }\}$. It follows from the non-permeable boundary condition (3.3) and $\tilde{v}\in C^1(\mathbb {T}_{q\pi }\times [-d,d])$ that $\tilde{v}=0$ on $\{\tilde{u}=\tilde{u}_{\max }\}$. Thus, $(\tilde{u}(x-\tilde{c}t,y),\tilde{v}(x-\tilde{c}t,y))$ is a shear flow, which is a contradiction.

Recalling Theorem 3.2, note that for $\beta \rightarrow 0$ in Theorem 3.11 we recover Proposition 3.3. The example in Remark 3.10 shows that for $\beta >0$ the speeds *c* of the zonally travelling waves need not be confined to *Ran*(*u*). In the following example, we construct such travelling waves near a Kolmogorov-type shear flow.

#### Example 3.12

Consider the region $\mathbb {T}_{2\pi }\times [-1,1]$ and the Kolmogorov shear flow $u(y)={\frac{1+\cos (\pi y)}{2}}$ for $y\in [-1,1]$, with $u_{\min }=0$ and $u_{\max }=1$. By Example 7.1 in [29], there exist travelling wave solutions

$$\begin{aligned} \vec {u}_n(x-c_nt,y)=(u_n(x-c_nt,y),v_n(x-c_nt,y)),\quad n\ge 1, \end{aligned}$$

to the equation (3.1) for $\beta >{9\over 16}\pi ^2$ such that $c_n\rightarrow c_0\in (-\frac{\beta }{\pi ^2}-\frac{1}{\pi ^2}\sqrt{\beta ^2+\pi ^2\beta },0)$, and

$$\begin{aligned} \Vert (u_n,v_n)-(u,0)\Vert _{C^2(\mathbb {T}_{2\pi }\times [-1,1])}\le C \Vert (u_n,v_n)-(u,0)\Vert _{H^5(\mathbb {T}_{2\pi }\times (-1,1))} \le {1\over n} \end{aligned}$$

by Corollary 2.6 in [29]. Thus

$$\begin{aligned} (u_n)_{\min }-\frac{2\beta }{\pi ^2}-\frac{2}{\pi ^2}\sqrt{\beta ^2+\pi ^2\beta ((u_n)_{\max }-(u_n)_{\min })}< -\frac{\beta }{\pi ^2}-\frac{1}{\pi ^2}\sqrt{\beta ^2+\pi ^2\beta }, \end{aligned}$$

$c_n<{c_0\over 2}$ and $(u_n)_{\min }>{c_0\over 2}$ for *n* sufficiently large, which implies

$$\begin{aligned} c_n\in \left( (u_n)_{\min }-\frac{2\beta }{\pi ^2}-\frac{2}{\pi ^2}\sqrt{\beta ^2+\pi ^2\beta ((u_n)_{\max }-(u_n)_{\min })},(u_n)_{\min }\right) . \end{aligned}$$

We now construct travelling waves, which are not small perturbations of shear flows, such that the wave speeds move away from the range of the zonal velocity to the left of its minimum as $\beta$ increases.

#### Example 3.13

Consider the region $\mathbb {T}_{2\pi }\times [-1,1]$. Let $\psi (x,y)=\alpha y+Y(x,y)$, with *Y* given in (3.25) and

$$\begin{aligned} \alpha =\frac{-\beta }{\frac{\pi ^2}{4}+1}-c. \end{aligned}$$

Then

$$\begin{aligned} \{\psi +cy,\gamma +\beta y\}=\left\{ (\alpha +c)y+Y,-\left( \frac{\pi ^2}{4}+1\right) Y+\beta y\right\} =0 \end{aligned}$$

so that $\psi (x-ct,y)$ is a non-sheared travelling wave solution of (3.4). Let $\beta _0=\frac{\pi }{2}(\frac{\pi ^2}{4}+1)$. Since

$$\begin{aligned} Ran(u)=Ran(-\psi _y)=&Ran\left( c+\frac{\beta }{\frac{\pi ^2}{4}+1}+\frac{\pi }{2}\cos (x)\sin \left( \frac{\pi y}{2}\right) \right) \\\nonumber =&\left[ c+\frac{\beta }{\frac{\pi ^2}{4}+1}-\frac{\pi }{2},c+\frac{\beta }{\frac{\pi ^2}{4}+1}+\frac{\pi }{2}\right] \,, \end{aligned}$$

we have:

(i) if $0 < \beta \le \beta _0$, then $c\in Ran(u)$ because

$$\begin{aligned}u_{\min }=c+\frac{\beta }{\frac{\pi ^2}{4}+1}-\frac{\pi }{2}\le c<c+\frac{\beta }{\frac{\pi ^2}{4}+1}+\frac{\pi }{2}=u_{\max }\;\Longrightarrow \;c\in Ran(u);\end{aligned}$$

(ii) if $\beta >\beta _0$, then $c<u_{\min }$ and

$$\begin{aligned} \begin{aligned}&u_{\min }-\frac{2\beta }{\pi ^2}-\frac{2}{\pi ^2}\sqrt{\beta ^2+\pi ^2\beta (u_{\max }-u_{\min })}\\ =&c+\frac{\beta }{\frac{\pi ^2}{4}+1}-\frac{\pi }{2}-\frac{2\beta }{\pi ^2}-\frac{2}{\pi ^2}\sqrt{\beta ^2+\pi ^3\beta }\\<&c+\frac{\beta }{\frac{\pi ^2}{4}+1}-\frac{\pi }{2}-\frac{4\beta }{\pi ^2}<c, \end{aligned} \end{aligned}$$

so that $c\in (u_{\min }-\frac{2\beta }{\pi ^2}-\frac{2}{\pi ^2}\sqrt{\beta ^2+\pi ^2\beta (u_{\max }-u_{\min })},u_{\min })$. In contrast to Example 3.12, this travelling wave solution $\psi (x-ct,y)$ is not a small perturbation of a shear flow since $\psi _x(x,y)=-\sin (x)\cos \left( \frac{\pi y}{2}\right)$.

We now establish that speeds of zonally travelling waves can be located to the left of the range $[u_{\min },u_{\max }]$ of the zonal velocity for any $\beta >0$ and any zonal period $T>0$. In particular, for steady flows, even if $\beta > 0$ is very small, regardless of the length of the period, non-sheared steady solutions satisfying (3.22) do exist. This is an interesting difference to the *f*-plane setting, given that Lemma 3.4 shows that any steady solution of (3.4) satisfying (3.22) has to be a shear flow.

#### Proposition 3.14

For any $\beta >0$, any $T>0$ and any $c\in \mathbb {R}$, there exists a non-sheared travelling wave solution $\psi (x-ct,y)\in C^\infty (\mathbb {T}_T\times [-d,d])$ of (3.4) with minimal zonal period *T* and speed $c<u_{\min }$. Consequently, non-sheared steady solutions satisfying (3.22) always exist.

#### Proof

Choose $\varepsilon >0$ such that $\beta >\varepsilon {\pi \over 2d}\left( {\pi ^2\over 4d^2}+{4\pi ^2\over T^2}\right)$. We define $\psi (x,y)=\alpha y+\varepsilon Y({2\pi \over T}x,{1\over d}y)$, where *Y* is given in (3.25) and

$$\begin{aligned} \alpha =\frac{-\beta }{{\pi ^2\over 4d^2}+{4\pi ^2\over T^2}}-c. \end{aligned}$$

Then the minimal zonal period of $\psi$ is *T*, $\psi _x(x,\pm d)=0$, and

$$\begin{aligned} \{\psi +cy,\gamma +\beta y\}=\left\{ (\alpha +c)y+\varepsilon Y,-\varepsilon \left( \frac{\pi ^2}{4d^2}+{4\pi ^2\over T^2}\right) Y+\beta y\right\} =0. \end{aligned}$$

Thus, $\psi (x-ct,y)$ is a non-sheared travelling wave solution of (3.4). Note that

$$\begin{aligned} Ran(u)=Ran(-\psi _y)=&Ran\left( c+\frac{\beta }{{\pi ^2\over 4d^2}+{4\pi ^2\over T^2}}+\varepsilon \frac{\pi }{2d}\cos \left( {2\pi \over T}x\right) \sin \left( \frac{\pi y}{2d}\right) \right) \\\nonumber =&\left[ c+\frac{\beta }{{\pi ^2\over 4d^2}+{4\pi ^2\over T^2}}-\varepsilon \frac{\pi }{2d},c+\frac{\beta }{{\pi ^2\over 4d^2}+{4\pi ^2\over T^2}}+\varepsilon \frac{\pi }{2d}\right] . \end{aligned}$$

Since $\beta >\varepsilon {\pi \over 2d}\left( {\pi ^2\over 4d^2}+{4\pi ^2\over T^2}\right)$, we have $c<u_{\min }$.

If $c=0$, since $u_{\min }>0$, we have $|\psi _y(x,y)|=|u(x,y)|=u(x,y)\ge u_{\min }>0$ for any $(x,y)\in \mathbb {T}_T\times [-d,d]$. Thus, the non-sheared steady solution $\psi (x,y)$ satisfies (3.22). $\square$

The above considerations show that, with respect to zonally propagating travelling waves, a marked difference between the *f*-plane and the $\beta$-plane is the possibility of propagation speeds $c<u_{\min }$. The investigation of such waves is eased by the following structural result.

#### Theorem 3.15

Let $\beta >0$. For any travelling wave solution

$$\begin{aligned} \vec {u}(x-ct,y)=(u(x-ct,y),v(x-ct,y))\in C^2(\mathbb {T}_{q\pi }\times [-d,d]) \end{aligned}$$

of the quasi-geostrophic equation (3.1) with $c < u_{\min }$, there exists some $G\in C^1(Ran(\psi +cy))$ such that

3.32 $$\begin{aligned} \Delta \psi +\beta y=G(\psi +cy). \end{aligned}$$

#### Proof

The proof is motivated by [15]. Define $\phi =\psi +cy$. Then

$$\begin{aligned} -\partial _y\phi =u-c\,,\quad \partial _x\phi =v\,, \end{aligned}$$

so that (3.4) can be rewritten as

3.33 $$\begin{aligned} -\partial _y\phi \partial _{x}(\gamma +\beta y)+\partial _{x}\phi \partial _{y}(\gamma +\beta y)=0. \end{aligned}$$

We change the variables (*x*, *y*) by setting

$$\begin{aligned} \tilde{q}=x\quad \text {and}\quad p=\phi (x,y). \end{aligned}$$

Then

$$\begin{aligned} {\partial {(\tilde{q},p)}\over \partial {(x,y)}}=\phi _y=c-u\ne 0,\quad \partial _x=\partial _{\tilde{q}}+\phi _x\partial _p,\quad \partial _y=\phi _y\partial _p, \end{aligned}$$

so that

$$\begin{aligned} \partial _{\tilde{q}}=\partial _x-{\phi _x\over \phi _y}\partial _y,\quad \partial _p={1\over \phi _y}\partial _y. \end{aligned}$$

By (3.33) we have

$$\begin{aligned} \partial _{\tilde{q}}(\gamma +\beta y)=\left( \partial _x-{\phi _x\over \phi _y} \partial _y\right) (\gamma +\beta y)=0. \end{aligned}$$

This, together with $\gamma \in C^1$ and $\phi \in C^3$, ensures the existence of a function $G\in C^1$ such that $\gamma +\beta y=G(p)=G(\phi )$ throughout the fluid. $\square$

We prove now a rigidity result that relies on (3.32).

#### Proposition 3.16

Let $\beta >0$. For a travelling wave solution $\psi (x-ct,y)\in C^3(\mathbb {T}_{q\pi }\times [-d,d])$ of the quasi-geostrophic vorticity equation (3.4) satisfying (3.32) with $G\in C^1(Ran(\psi +cy))$, if the minimal zonal period is ${q\pi \over k_0}$ (with $k_0 \ge 1$ an integer) and $-G'<{\pi ^2\over 4d^2}+{4k_0^{2}\over q^2}$, then $\psi (x-ct,y)$ is a shear flow.

#### Proof

Taking the derivative of (3.32) with respect to the *x*-variable, we obtain

$$\begin{aligned} \Delta \psi _x=G'(\psi +cy)\psi _x. \end{aligned}$$

Let $X=\left\{ \phi \in H^1\left( \mathbb {T}_{q\pi \over k_0}\times [-d,d]\right) \bigg |\phi (x,\pm d)=0\right\}$. We decompose the eigenvalue problem

3.34 $$\begin{aligned} \left\{ \begin{aligned}&-\Delta \phi =\lambda \phi ,\\&\phi \in X, \end{aligned} \right. \end{aligned}$$

into a sequence of boundary-value problems for ordinary differential equations, namely

3.35 $$\begin{aligned} \left\{ \begin{aligned}&-\phi _0''=\lambda \phi _0,\\&\phi _0\in H_0^1(-d,d), \end{aligned} \right. \end{aligned}$$

and

3.36 $$\begin{aligned} \left\{ \begin{aligned}&-\phi _k''+\left( {2kk_0\over q}\right) ^2\phi _k=\lambda \phi _k,\\&\phi _k\in H_0^1(-d,d), \end{aligned} \right. \end{aligned}$$

where $k\ne 0$. The eigenvalues of (3.35) are denoted by $\frac{\pi ^2}{4d^2}=\lambda _{1}^{(0)}<\lambda _{2}^{(0)}<\cdots<\lambda _{j}^{(0)}<\cdots$. They diverge to $+\infty$. The principal eigenvalue of (3.36) for $k=1$ is $\frac{\pi ^2}{4d^2}+{4k_0^2\over q^2}$. The eigenvalues of (3.35) less than $\frac{\pi ^2}{4d^2}+{4k_0^2\over q^2}$ are denoted by $\lambda _{1}^{(0)}<\cdots <\lambda _{j_0}^{(0)}$ with corresponding eigenfunctions denoted by $\phi _{1}^{(0)},\cdots ,\phi _{j_0}^{(0)}$. Since the $(j_0+1)$-th eigenvalue of (3.34) is $\lambda _{j_0+1}=\frac{\pi ^2}{4d^2}+{4k_0^2\over q^2}$, we have

$$\begin{aligned} \frac{\pi ^2}{4d^2}+{4k_0^2\over q^2}=\lambda _{j_0+1}=\inf _{\phi \bot _{L^2}span\{\phi _{1}^{(0)},\cdots ,\phi _{j_0}^{(0)}\},\;\phi \in X} \frac{\int _{-d}^{d}\int _{0}^{\frac{q\pi }{k_0}}|\nabla \phi |^2dxdy}{\int _{-d}^{d}\int _{0}^{\frac{q\pi }{k_0}}|\phi |^2dxdy}. \end{aligned}$$

Since

$$\begin{aligned} \int _{-d}^{d}\int _{0}^{\frac{q\pi }{k_0}}\psi _x\phi _{j}^{(0)}(y)dxdy=0,\;1\le j\le j_0, \end{aligned}$$

we must have

3.37 $$\begin{aligned} \int _{-d}^{d}\int _{0}^{\frac{q\pi }{k_0}}|\nabla \psi _{x}|^2dxdy \ge \left( \frac{\pi ^2}{4d^2}+{4k_0^2\over q^2}\right) \int _{-d}^{d}\int _{0}^{\frac{q\pi }{k_0}}|\psi _{x}|^2dxdy. \end{aligned}$$

If $\psi _{x}\not \equiv 0$, we obtain the contradiction by

$$\begin{aligned}&\left( \frac{\pi ^2}{4d^2}+{4k_0^2\over q^2}\right) \int _{-d}^{d}\int _{0}^{\frac{q\pi }{k_0}}|\psi _{x}|^2dxdy \le \int _{-d}^{d}\int _{0}^{\frac{q\pi }{k_0}}|\nabla \psi _{x}|^2dxdy\\ =&\int _{-d}^{d}\int _{0}^{\frac{q\pi }{k_0}}-\psi _x\Delta \psi _{x}dxdy=\int _{-d}^{d}\int _{0}^{\frac{q\pi }{k_0}}-G'(\psi +cy)|\psi _{x}|^2dxdy\\ <&\int _{-d}^{d}\int _{0}^{\frac{q\pi }{k_0}}\left( \frac{\pi ^2}{4d^2}+{4k_0^2\over q^2}\right) |\psi _{x}|^2dxdy. \end{aligned}$$

Consequently, $\psi _x\equiv 0$ and $\psi (x-ct,y)$ is a shear flow. $\square$

#### Remark 3.17

Proposition 3.16 is optimal in the sense that for any $\beta >0$, any $c\in \mathbb {R}$ and any $k_0\ge 1$, there exist non-sheared travelling wave solutions $\psi (x-ct,y)$ satisfying (3.32), where $-G'={\pi ^2\over 4d^2}+{4k_0^{2}\over q^2}$ and the minimal period of $\psi$ in the *x*-direction is ${q\pi \over k_0}$. Indeed, the non-sheared travelling wave solutions $\psi (x-ct,y)$ can be constructed as follows. Consider the eigenvalue problem (3.34). It is clear that ${\pi ^2\over 4d^2}+{4k_0^{2}\over q^2}$ is an eigenvalue of (3.34) with a corresponding eigenfunction $\cos \left( {2k_0\over q}x\right) \cos \left( {\pi \over 2d}y\right)$. Let $\psi (x,y)=\alpha y+\cos \left( {2k_0\over q}x\right) \cos \left( {\pi \over 2d}y\right)$ with $\alpha =-{\beta \over {\pi ^2\over 4d^2}+{4k_0^{2}\over q^2}}-c$. Then

$$\begin{aligned} \{\psi +cy,\omega +\beta y\} =&\{(\alpha +c)y+\cos \left( {2k_0\over q}x\right) \cos \left( {\pi \over 2d}y\right) ,\\&-\left( {\pi ^2\over 4d^2}+{4k_0^{2}\over q^2}\right) \cos \left( {2k_0\over q}x\right) \cos \left( {\pi \over 2d}y\right) +\beta y\}=0 \end{aligned}$$

and thus $\psi (x-ct,y)$ is a non-sheared travelling wave solution of (3.4).

#### Remark 3.18

In the research literature, there are some related rigidity results based on the elliptic equation (3.32) and maximum principles.

(1) Let $\beta =0$. For a travelling wave solution $\vec {u}(x-ct,y)=(u(x-ct,y),v(x-ct,y))$ of (3.1), it was shown in [28] that if $u>c$, then $\vec {u}(x-ct,y)$ is a shear flow. The proof in [28] consists in first reformulating the elliptic equation (3.32) by means of a hodograph transformation and then using a maximum principle. The hodograph change of variables was introduced in [19] and has been used to transform the free-boundary problem to a boundary problem in a fixed domain in studies of the symmetry of gravity surface water-waves [9, 25]. These symmetry results rely on the fact that the pressure is constant on the streamline representing the free surface of a travelling water wave. This property cannot be expected for the astrophysical flows considered in the present paper, where asymmetry prevails (see the next subsection). Let us note that Proposition 3.3 not only improves the result in [28] but also has an elementary proof, based on simple calculus.

(2) With the help of the elliptic equation (3.32), we can present an alternative proof of Theorem 3.2 by applying the following Liouville Theorem in [16], slightly modified to better suit our purposes: if $\Omega =\mathbb {T}_{q\pi }\times [-d,d]$, and if ${{\mathfrak {g}}}={{\mathfrak {g}}}(y,\phi )$ is a Lipschitz function and $\phi \in C^2(\Omega )$ is a solution to

$$\begin{aligned} \Delta \phi +{{\mathfrak {g}}}(y,\phi )=0 \quad \text {in}\quad \Omega , \end{aligned}$$

where $\phi (x,-d)=0$ and $\phi (x,d)=C>0$ for $x\in \mathbb {T}_{q\pi }$, then $\phi$ is independent of *x* if ${{\mathfrak {g}}}_y\ge 0$ and if $\phi _y\ge 0$ on $\Omega$. Indeed, since $c>u_{\max }$, by the same argument used in the proof of Theorem 3.15, we obtain (3.32). Defining $\phi (x,y)=\psi (x,y)+cy-\psi (0,-d)+cd$ for $(x,y)\in \Omega$, then $\phi$ solves the elliptic equation

$$\begin{aligned} \Delta \phi +\beta y-G(\phi +\psi (0,-d)-cd)=0, \end{aligned}$$

and $\phi (x,-d)=0$. The assumption $c>u_{\max }$ ensures $\phi _y=\psi _y+c>0$ and $\phi (\cdot ,d)>0$. Setting ${\mathfrak g}(y,\phi )=\beta y-G(\phi +\psi (0,-d)-cd)$, we now have ${\mathfrak g}_y=\beta \ge 0$ and the above Liouville Theorem yields that $\phi$ and $\psi$ are independent of *x*.

The functional *G* in (3.32) is not necessarily invertible. However, this property holds if $\beta$ is not in the range of $-\partial _y\gamma$.

#### Lemma 3.19

For a travelling wave solution $\vec {u}(x-ct,y)\in C^2(\mathbb {T}_{q\pi }\times [-d,d])$ of (3.1) with $\beta \notin Ran(-\partial _y\gamma )$, there exists $F\in C^1(Ran(\gamma +\beta y))$ such that

3.38 $$\begin{aligned} \psi +cy=F(\gamma +\beta y)=F(\Delta \psi +\beta y). \end{aligned}$$

#### Proof

We change the variables (*x*, *y*) to be

$$\begin{aligned} \tilde{q}=x\quad \text {and}\quad p=\gamma (x,y)+\beta y. \end{aligned}$$

Then $${\partial {(\tilde{q},p)}\over \partial {(x,y)}}=\partial _y\gamma +\beta \ne 0$$,

$$\begin{aligned} \partial _x=\partial _{\tilde{q}}+\partial _x\gamma \partial _p\quad \text {and}\quad \partial _y=(\partial _y\gamma +\beta )\partial _p. \end{aligned}$$

Thus, $\partial _{\tilde{q}}=\partial _x-\partial _x\gamma {\partial _y\over \partial _y\gamma +\beta }$ and $\partial _p={1\over \partial _y\gamma +\beta }\partial _y$. By (3.23) we have

$$\begin{aligned} \partial _{\tilde{q}}(\psi +cy)=\left( \partial _x-\partial _x\gamma {\partial _y\over \partial _y\gamma +\beta }\right) (\psi +cy)=0. \end{aligned}$$

This, together with $\gamma \in C^1$ and $\psi \in C^3$, implies that there exists a function $F\in C^1$ such that $\psi +cy=F(p)=F(\gamma +\beta y)$ throughout the fluid. $\square$

We prove now a rigidity result that relies on (3.38).

#### Proposition 3.20

Let $\beta \ge 0$. For a travelling wave solution $\psi (x-ct,y)\in C^3(\mathbb {T}_{q\pi }\times [-d,d])$ of the quasi-geostrophic vorticity equation (3.4) satisfying (3.38) with $F\in C^1(Ran(\gamma +\beta y))$, if the minimal zonal period is ${q\pi \over k_0}$ (with $k_0 \ge 1$ an integer), and *F* satisfies $F'\ge 0$ or $F'< -{1/ \left( {\pi ^2\over 4d^2}+{4k_0^{2}\over q^2}\right) }$, then $\psi (x-ct,y)$ is a shear flow.

#### Proof

The proof follows from Proposition 3.16 if the inverse of *F* is in $C^1$. However, since *F* is not invertible in general, we have to argue differently.

Taking the derivative of (3.38) with respect to the *x*-variable, we obtain

3.39 $$\begin{aligned} \psi _x=F'(\Delta \psi +\beta y)\Delta \psi _x. \end{aligned}$$

Since the minimal zonal period of $\psi _x$ is ${q\pi \over k_0}$ and its zero’th Fourier mode is 0, by (3.37) we have

$$\begin{aligned} \int _{-d}^{d}\int _{0}^{\frac{q\pi }{k_0}}|\nabla \psi _{x}|^2dxdy=&-\int _{-d}^{d}\int _{0}^{\frac{q\pi }{k_0}}\psi _x\Delta \psi _{x}dxdy\\ \le&\left( \int _{-d}^{d}\int _{0}^{\frac{q\pi }{k_0}}|\psi _x|^2 dxdy\right) ^{1\over 2}\left( \int _{-d}^{d}\int _{0}^{\frac{q\pi }{k_0}}|\Delta \psi _{x}|^2 dxdy\right) ^{1\over 2}\\ \le&{1\over \sqrt{\frac{\pi ^2}{4d^2}+{4k_0^2\over q^2}}}\left( \int _{-d}^{d}\int _{0}^{\frac{q\pi }{k_0}}|\nabla \psi _{x}|^2dxdy\right) ^{1\over 2}\left( \int _{-d}^{d}\int _{0}^{\frac{q\pi }{k_0}}|\Delta \psi _{x}|^2 dxdy\right) ^{1\over 2}. \end{aligned}$$

This proves

$$\begin{aligned} \int _{-d}^{d}\int _{0}^{\frac{q\pi }{k_0}}|\nabla \psi _{x}|^2dxdy \le&{1\over \frac{\pi ^2}{4d^2}+{4k_0^2\over q^2}}\int _{-d}^{d}\int _{0}^{\frac{q\pi }{k_0}}|\Delta \psi _{x}|^2 dxdy. \end{aligned}$$

Multiplying (3.39) by $\Delta \psi _{x}$, integrating over $\mathbb {T}_{\frac{q\pi }{k_0}}\times [-d,d]$ and performing integration by parts give

3.40 $$\begin{aligned} \int _{-d}^{d}\int _{0}^{\frac{q\pi }{k_0}}-|\nabla \psi _{x}|^2dxdy=\int _{-d}^{d}\int _{0}^{\frac{q\pi }{k_0}}F'(\Delta \psi +\beta y)|\Delta \psi _x|^2dxdy. \end{aligned}$$

If $F'\ge 0$, then it follows from (3.40) and $\psi _x(\cdot ,\pm d)=0$ that $\psi _x=0$ on $\mathbb {T}_{\frac{q\pi }{k_0}}\times [-d,d]$. Thus $\psi (x-ct,y)$ is a shear flow.

Now we consider the case $F'< -{1/ \left( {\pi ^2\over 4d^2}+{4k_0^{2}\over q^2}\right) }$. If $\Delta \psi _{x}\not \equiv 0$, we obtain the contradiction by

$$\begin{aligned}&-{1\over \frac{\pi ^2}{4d^2}+{4k_0^2\over q^2}}\int _{-d}^{d}\int _{0}^{\frac{q\pi }{k_0}}|\Delta \psi _{x}|^2 dxdy \le -\int _{-d}^{d}\int _{0}^{\frac{q\pi }{k_0}}|\nabla \psi _{x}|^2dxdy\\ =&\int _{-d}^{d}\int _{0}^{\frac{q\pi }{k_0}}F'(\Delta \psi +\beta y)|\Delta \psi _x|^2dxdy<-{1\over \frac{\pi ^2}{4d^2}+{4k_0^2\over q^2}}\int _{-d}^{d}\int _{0}^{\frac{q\pi }{k_0}}|\Delta \psi _{x}|^2 dxdy. \end{aligned}$$

Consequently, $\Delta \psi _x=0$, $\nabla \psi _x=\vec {0}$ and $\psi (x-ct,y)$ is a shear flow. $\square$

#### Remark 3.21

For $k_0=1$, the conditions on *F* in Proposition 3.20 are the celebrated Arnol’d stability conditions, and a rigidity result ensuring that such steady flows must be shear flows is often referred to as an Andrews-type theorem [3, 5, 6, 41].

Finally, we discuss the real spectra of the linearized operator around a shear flow, which turns out to be fundamentally different from the spherical case, see Remark 4.3. For a shear flow with vorticity $\gamma _0(y)=\Delta \psi _0(y)\in C^1([-d,d])$, the linearized equation around $\gamma _0(y)$ is

3.41 $$\begin{aligned} \partial _{t}\gamma =\psi _0'\partial _{x}\gamma -(\gamma _0'+\beta )\partial _{x}\Delta ^{-1}\gamma . \end{aligned}$$

Let $\gamma (x,y)=\sum _{k\ne 0}\gamma _k(y)e^{ik\alpha x}$ and $\psi (x,y)=\sum _{k\ne 0}\psi _k(y)e^{ik\alpha x}$, where $\alpha ={2\over q}$. Decomposing (3.41) into the Fourier modes, we have

$$\begin{aligned}\partial _{t}\gamma _k=-ik\alpha \mathbb {L}_{k\alpha ,\beta }\gamma _k,\end{aligned}$$

where

3.42 $$\begin{aligned} \mathbb {L}_{k\alpha ,\beta }\gamma _k=-\psi _{0}'\gamma _k+(\gamma _0'+\beta )\left( {d^2\over dy^2}-(k\alpha )^2\right) ^{-1}\gamma _k \end{aligned}$$

is the linearized operator for the *k*’th mode acting on $L^2(-d,d)$, and

$$\begin{aligned} \gamma _k=\left( {d^2\over dy^2}-(k\alpha )^2\right) \psi _k. \end{aligned}$$

Since $\mathbb {L}_{k\alpha ,\beta }$ is a compact perturbation of the multiplication operator $-\psi _{0}'$, its essential spectra is $\sigma _{ess} (\mathbb {L}_{k\alpha ,\beta })=Ran(-\psi _{0}')$. On the other hand, if $c\notin Ran(-\psi _{0}')$ is an eigenvalue of $\mathbb {L}_{k\alpha ,\beta }$, then

$$\begin{aligned}(\psi _{0}'+c)\gamma _k-\psi _k(\gamma _0'+\beta )=0,\end{aligned}$$

which gives the Rayleigh equation

3.43 $$\begin{aligned} \psi _k''-(k\alpha )^2\psi _k-\frac{\gamma _0'+\beta }{\psi _0'+c}\psi _k=0, \quad \psi _k(\pm d)=0. \end{aligned}$$

We call $(c,k,\beta ,\psi )$ with $c \in \mathbb {R}$ a *neutral mode* if $\psi \in H^2(-d,d)$ is a non-trivial solution of (3.43). We characterize now the real spectra of the linearized operator $\mathbb {L}_{k\alpha ,\beta }$ around the shear flow $\psi _0$.

Intuitively, motivated by the following lemma, the rigidity of traveling waves near a shear flow might be expected. It turns actually out that the rigidity results in Theorems 3.2 and 3.11 apply for travelling waves with arbitrarily large meridional velocity, whereas a travelling wave that is a small perturbation of a shear flow necessarily has a small meridional velocity.

#### Lemma 3.22

Let $\psi _0\in C^3([-d,d])$ be a shear flow and let $k\ne 0$ be an integer. Then

(i) if $$\beta =0$$, then $$\sigma (\mathbb {L}_{k\alpha ,\beta })\cap \mathbb {R}=Ran(-\psi _{0}')$$;

(ii) if $\beta >0$, then $\sigma (\mathbb {L}_{k\alpha ,\beta })\cap \mathbb {R}\subset \big [c_\beta ,$ $(-\psi _0')_{\max }\big ]$, where

$$\begin{aligned} c_\beta \triangleq (-\psi _0')_{\min }-\frac{2\beta d^2}{\pi ^2}-\frac{2d^2}{\pi ^2}\sqrt{\beta ^2+{\pi ^2\beta \over d^2}((-\psi _0')_{\max }-(-\psi _0')_{\min })}<(-\psi _0')_{\min }. \end{aligned}$$

In terms of the neutral modes, Lemma 3.22 can be restated as follows.

**Restatement of Lemma** 3.22. Let $\psi _0\in C^3([-d,d])$ be a shear flow and let $k\ne 0$ be an integer. Then

(i) if $\beta =0$, then for any neutral mode $(c,k,\beta ,\psi )$, *c* must lie in $Ran(-\psi _{0}')$;

(ii) if $\beta >0$, then for any neutral mode $(c,k,\beta ,\psi )$, *c* must lie in $\big [c_\beta ,$ $(-\psi _0')_{\max }\big ]$.

#### Proof

The proof is based on ODE analysis and is motivated by [33, 34]. We give it here for completeness.

Let $c\notin Ran(-\psi _{0}')$. If $c\in \sigma (\mathbb {L}_{k\alpha ,\beta })\cap \mathbb {R}$, then there exists $0\ne \psi \in H^2(-d,d)$ such that $\mathbb {L}_{k\alpha ,\beta }\psi =c\psi$. For $y\in [-d,d]$, we define

$$\begin{aligned} H(y)=\frac{\psi (y)}{-\psi _{0}'(y)-c}.\end{aligned}$$

Then

$$\begin{aligned} H'=\frac{-\psi '(\psi _0'+c)+\psi \psi _0''}{(\psi _{0}'+c)^2}. \end{aligned}$$

Thus,

$$\begin{aligned} ((\psi _{0}'+c)^2H')'=-\psi ''(\psi _0'+c)+\psi \psi _0'''. \end{aligned}$$

Since $\mathbb {L}_{k\alpha ,\beta }\psi =c\psi$, $\psi$ solves (3.43). Then

3.44 $$\begin{aligned} ((\psi _{0}'+c)^2H')'=&\left( -(k\alpha )^2\psi -\frac{\gamma _0'+\beta }{\psi _0'+c}\psi \right) (\psi _0'+c)+\psi \psi _0''' =(k\alpha )^2(\psi _{0}'+c)^2H-\beta \psi . \end{aligned}$$

Multiplying (3.44) by *H*, integrating over $[-d,d]$ and performing integration by parts give

3.45 $$\begin{aligned} \int _{-d}^{d}\left( (\psi _0'+c)^2|H'|^2+(k\alpha )^2(\psi _0'+c)^2 |H|^2\right) dy=\int _{-d}^d-\beta (\psi _0'+c) |H|^2 dy. \end{aligned}$$

First, we prove part (i) of the statement. If $\beta =0$, then $H=0$ by (3.45). Thus $\psi =0$, which is a contradiction.

We now prove part (ii) of the statement. If $\beta >0$ and $c>(-\psi _0')_{\max }$, then $\psi _0'(y)+c>0$ for $y\in [-d,d]$. Then $H=0$ by (3.45). Thus, $\psi =0$, which is a contradiction.

Now, we assume that $c<c_\beta =(-\psi _0')_{\min }-\frac{2\beta d^2}{\pi ^2}-\frac{2d^2}{\pi ^2}\sqrt{\beta ^2+{\pi ^2\beta \over d^2}((-\psi _0')_{\max }-(-\psi _0')_{\min })}$. Since $c<(-\psi _0')_{\min }$ we have $|\psi _0'+c|=-\psi _0'-c\ge (-\psi _0')_{\min }-c>0$. Thus

$$\begin{aligned} \text {Left hand side of } (3.45)\ge ((-\psi _0')_{\min }-c)^2 \int _{-d}^{d}|H'|^2 dy. \end{aligned}$$

Combining with the Poincaré inequality

$$\begin{aligned} \int _{-d}^d|H'|^2dy\ge {\pi ^2\over 4d^2}\int _{-d}^d|H|^2dy, \end{aligned}$$

we have

$$\begin{aligned} \int _{-d}^d-\beta (\psi _0'+c) |H|^2 dy&\ge ((-\psi _0')_{\min }-c)^2 \int _{-d}^{d}|H'|^2 dy \\  &\ge ((-\psi _0')_{\min }-c)^2{\pi ^2\over 4d^2}\int _{-d}^d|H|^2dy. \end{aligned}$$

Thus

$$\begin{aligned} \beta \left( (-\psi _0')_{\max }-c\right) \int _{-d}^d |H|^2 dy\ge ((-\psi _0')_{\min }-c)^2{\pi ^2\over 4d^2}\int _{-d}^d|H|^2dy. \end{aligned}$$

Since $c<(-\psi _0')_{\min }-\frac{2\beta d^2}{\pi ^2}-\frac{2d^2}{\pi ^2}\sqrt{\beta ^2+{\pi ^2\beta \over d^2}((-\psi _0')_{\max }-(-\psi _0')_{\min })}$, we have

$$\begin{aligned} \beta \left( (-\psi _0')_{\max }-c\right) -((-\psi _0')_{\min }-c)^2{\pi ^2\over 4d^2}<0. \end{aligned}$$

Thus

$$\begin{aligned} \int _{-d}^d |H|^2 dy=0. \end{aligned}$$

Then $H=0$ so that $\psi =0$, which is a contradiction. $\square$

### Asymmetric travelling wave solutions for 2D quasi-geostrophic equation

Saturn’s circumpolar hexagon is a zonally symmetric travelling wave (see the discussion in [7]) but most observed wave patterns in the visible atmospheres of the outer planets do not exhibit zonal symmetry [26, 32]. In this subsection, we construct some examples of steady flows and travelling wave solutions of the quasi-geostrophic vorticity equation (3.4) that are not symmetric, up to any translation, in the *x*-direction. That is, there is no $x_0\in \mathbb {T}_{q\pi }$ such that all the streamlines are symmetric about the line $\{x=x_0\}$.

Generally speaking, the superposition of two travelling wave solutions is not necessarily another travelling wave solution. However, by choosing an appropriate region and by constructing two travelling wave solutions with opposite parity properties in the *x*-direction, which correspond to the same eigenvalue of the Laplace operator in this region, their superposition can be again a travelling wave solution. As we shall see, these travelling waves are zonally asymmetric.

#### Example 3.23

Consider the flow in the finite channel $(x,y)\in \mathbb {T}_{2\pi }\times \left[ -{\pi \over 2},{\pi \over 2}\right]$. Then

$$\begin{aligned}Y(x,y)=\cos (3x)\cos (y)+\sin (x)\cos (3y)\end{aligned}$$

solves the eigenvalue problem

$$\begin{aligned} {\left\{ \begin{array}{ll} -\Delta Y=10 Y\quad \textrm{on}& \quad \mathbb {T}_{2\pi }\times \left[ -{\pi \over 2},{\pi \over 2}\right] ,\\ \quad Y=0\quad \quad \;\;\,\,\textrm{on}&  \quad \; y=\pm {\pi \over 2}. \end{array}\right. } \end{aligned}$$

Let $\beta \ge 0$ and $c\in \mathbb {R}$. With $\alpha =\frac{-\beta }{10}-c$, set

$$\begin{aligned} \psi (x,y)=\alpha y+Y(x,y)=\alpha y+\cos (3x)\cos (y)+\sin (x)\cos (3y). \end{aligned}$$

Then

$$\begin{aligned} \{\psi +cy,\omega +\beta y\}=\left\{ \left( \frac{-\beta }{10}-c\right) y+Y+cy,-10Y+\beta y\right\} =0. \end{aligned}$$

Since $\psi _{x}\left( x,\pm {\pi \over 2}\right) =0$ for $x\in \mathbb {T}_{2\pi }$, $\psi$ satisfies the non-permeable boundary condition, with $\psi (x-ct,y)$ a non-sheared travelling wave solution of (3.4). We now construct asymmetric steady flows and travelling wave solutions.

(i) **Zonally asymmetric cat’s eyes steady flow in the** *f*-**plane setting.**

**Fig. 2:**
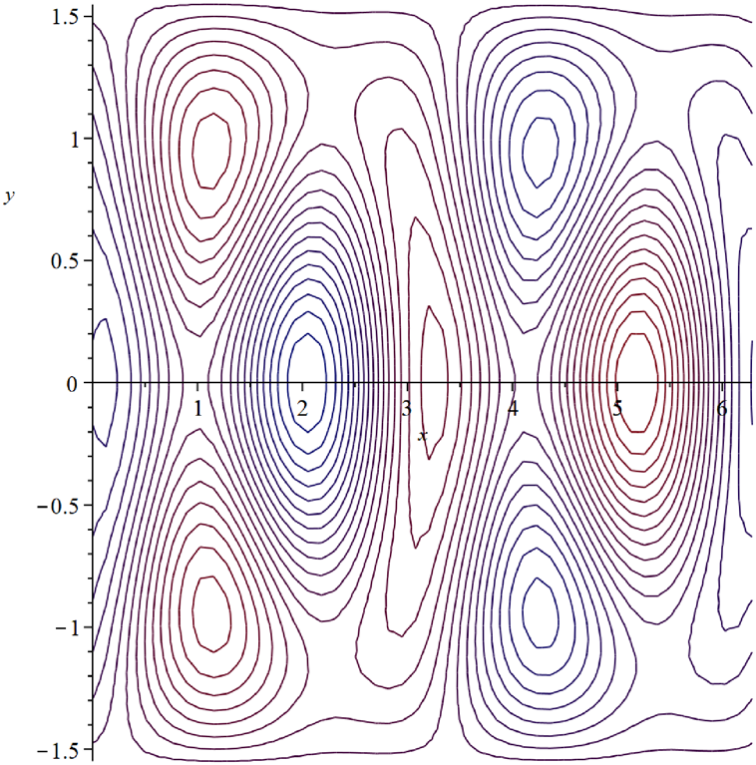
Zonally asymmetric cat’s eyes flow in the *f*-plane setting

Let $\beta =c=0$. Then $\alpha =0$ and $\psi (x,y)=Y(x,y)$ is a steady solution of (3.4), whose speed $c=0$ lies in the interior $(Ran(-\psi _y))^{\circ }$ of the set $Ran(-\psi _y)$. The streamlines are depicted in Fig. 2, showing the lack of symmetry in the *x*-direction.

(ii) **Zonally asymmetric cat’s eyes steady flow in the** $\beta$-**plane setting.**

**Fig. 3:**
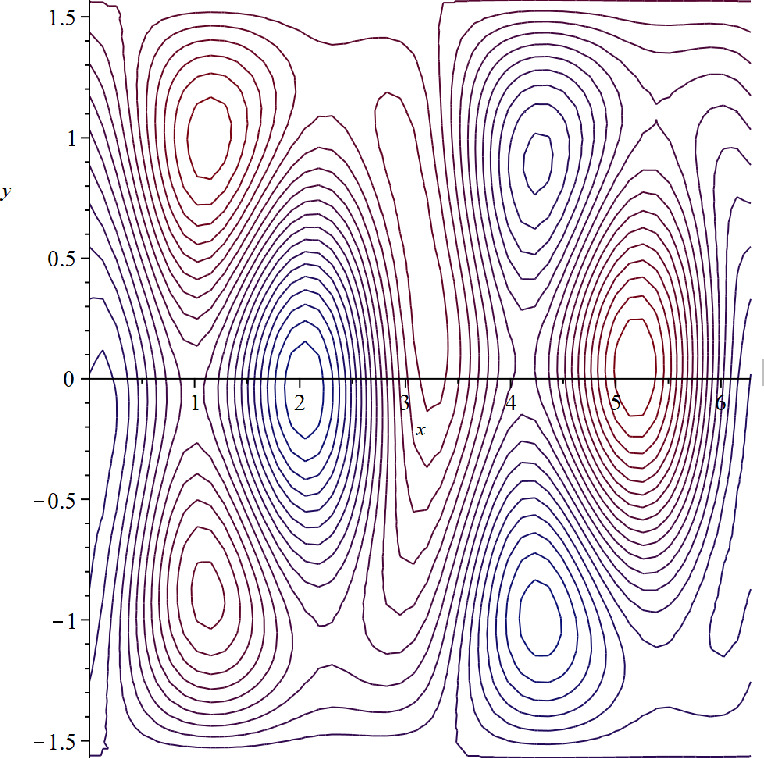
Zonally asymmetric cat’s eyes flow in the $\beta$-plane setting

Let $\beta =4$ and $c=0$. Then $\alpha =-0.4$, $c=0\in (Ran(-\psi _y))^{\circ }$, and

$$\begin{aligned} \psi (x,y)=-0.4y+Y(x,y) \end{aligned}$$

is a zonally asymmetric steady solution of (3.4), whose streamlines are depicted in Fig. 3.

(iii) **Zonally asymmetric unidirectional steady flow in the** $\beta$-**plane setting.**

**Fig. 4:**
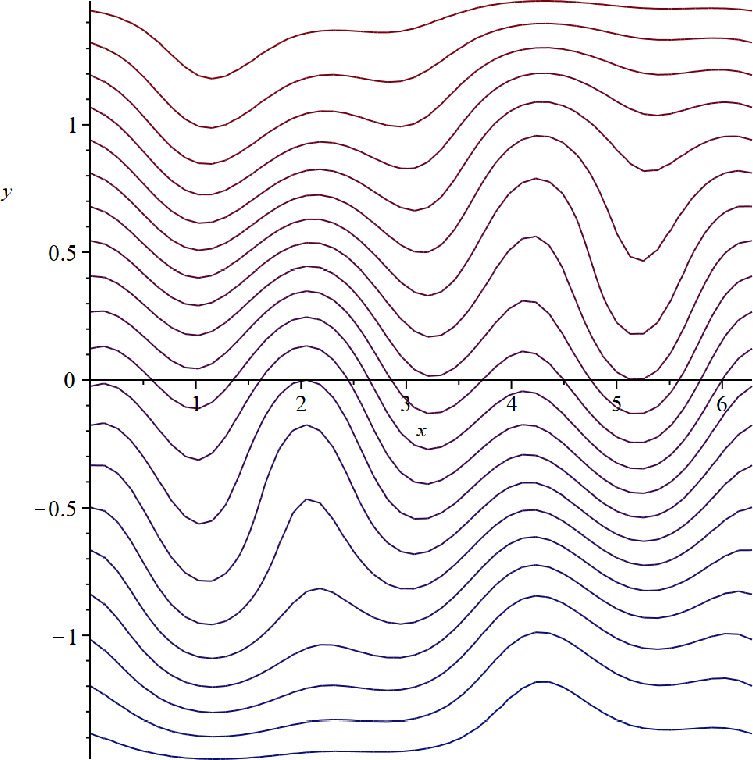
Zonally asymmetric unidirectional steady flow in the $\beta$-plane setting

Let $\beta =50, c=0$. Then $\alpha =-5$ and $\psi (x,y)=-5y+Y(x,y)$ is a steady solution of (3.4). Since $Y_y\in [-4,4]$ and $\psi _y(x,y)=-5+Y_y(x,y)$, we have $c=0<(-\psi _y)_{\min }$, so that the zonally asymmetric streamlines have no cat’s eyes structure and move in one direction (see Fig. 4).

One can similarly construct zonally asymmetric cat’s eyes and unidirectional travelling waves in the $\beta$-plane setting, as well as asymmetric cat’s eyes travelling waves in the *f*-plane setting. Note that by Proposition 3.7, any unidirectional travelling wave must be a shear flow in the *f*-plane setting.

We give now some simple examples showing the symmetry-breaking effect of the $\beta$-plane. This symmetry-breaking phenomenon also manifests itself in the generalization of Stuart’s planar cat’s eyes flow in [40] to the $\beta$-plane (see [31]). While viscosity also brings about a loss of symmetry for the planar Stuart vortices [20], the viscous setting is not relevant for stratospheric flows. In this context, note that the existence of Stuart vortices on a fixed sphere is established [17], but for a rotating sphere no such exact solutions are known (see [8] for a perturbative approach).

**Fig. 5:**
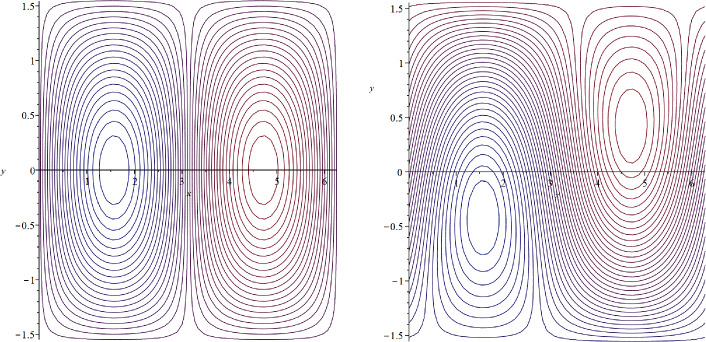
Streamlines of the steady solution (3.46) for $\beta =0$ (left) and for $\beta =0.8$ (right)

#### Example 3.24

Consider the flow in the finite channel $(x,y)\in \mathbb {T}_{2\pi }\times \left[ -{\pi \over 2},{\pi \over 2}\right]$.

(i) **Symmetry-breaking in the** *y*-**direction due to the** $\beta$-**plane effect.** One can check that

3.46 $$\begin{aligned} \psi (x,y)= -\frac{\beta }{2}\,y+Y(x,y) \end{aligned}$$

is a steady solution of (3.4) if $Y(x,y)=\cos (y)\sin (x)$. The streamlines for $\beta =0$ and for $\beta =0.8$, depicted in Fig. 5, show that the $\beta$-plane effect induces symmetry-breaking in the *y*-direction. Note that the steady solution in Example 3.23 also illustrates the symmetry-breaking $\beta$-plane effect in the *y*-direction, the solution being symmetric in the *y*-direction for $\beta =0$ (Fig. 2), but asymmetric in the *y*-direction for $\beta =4$ and for $\beta = 50$ (see Figs. 3 and 4).

(ii) **Symmetry-breaking in a global sense due to the** $\beta$-**plane effect.** Note that

**Fig. 6:**
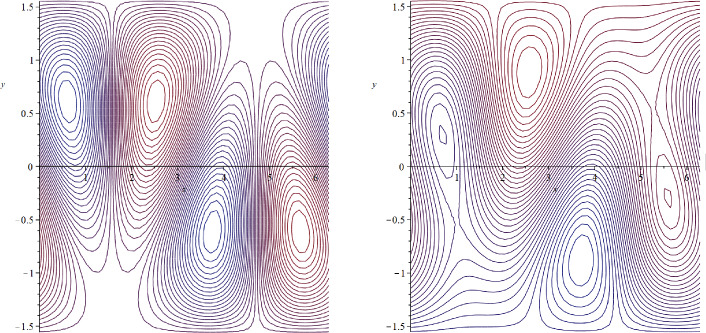
Streamlines of the steady solution (3.47) for $\beta =0$ (left) and for $\beta =5$ (right). While the pattern is asymmetric in the *x*-direction even for $\beta =0$, the level set $\{\psi =0\}$ is nevertheless symmetric in this direction. However, two cat’s eyes become “smaller" and the other two become “bigger" for $\beta =5$, showing that the symmetry is globally broken

3.47 $$\begin{aligned} \psi (x,y)=-\frac{\beta }{5}\, y+Y(x,y) \end{aligned}$$

is a steady solution of (3.4) if $Y(x,y)=\sin (2y)\cos (x)+\cos (y)\sin (2x)$. The streamline pattern (see Fig. 6) shows the global symmetry-breaking due to the $\beta$-plane effect.

## The Euler Equation on the 2-Sphere

In this section we study stratospheric travelling waves on a rotating sphere. For the rigidity of travelling waves, it turns out that there are some fundamental differences between the spherical geometry and the flat-space geometry of the *f*-plane or that of the $\beta$-plane setting. In the flat-space geometry, whether $\beta =0$ or not affects the wave speeds of the genuine travelling waves. In contrast to this, even if we only consider traveling waves near a zonal flow, the case $\omega =0$ is not distinguished since only the relative position between $-\omega$ and the essential spectra of the linearized operator around the background zonal flow is important (see Remark 4.3 for details). Other than the rigidity of travelling waves near a zonal flow, in this section we also obtain some rigidity results that rely on the vorticity-stream function relationships valid on a rotating sphere. Furthermore, we construct asymmetric travelling waves and present a symmetry-breaking phenomenon due to the rotation of the sphere.

The Euler equation on the 2-sphere $\mathbb {S}^2$, with standard metric, in a frame rotating at rate $\omega >0$ about the polar axis, can be written as

4.1 $$\begin{aligned} \partial _t\Delta _{\mathbb {S}^2}\psi +{1\over \cos (\theta )}[-\partial _\theta \psi \partial _\varphi +\partial _\varphi \psi \partial _\theta ](\Delta _{\mathbb {S}^2} \psi +2\omega \sin (\theta ))=0, \end{aligned}$$

in terms of the stream function $\psi$ defined by

$$\begin{aligned} u=-\partial _\theta \psi , \qquad v=\frac{\partial _\varphi \psi }{\cos \theta }, \end{aligned}$$

where $(\varphi ,\theta )\in \mathbb {T}_{2\pi }\times (-{\pi \over 2},{\pi \over 2})$ and the Laplace-Beltrami operator is defined by

$$\begin{aligned} \Delta _{\mathbb {S}^2}\psi =\partial _\theta ^2\psi -\tan (\theta )\partial _\theta \psi +{1\over \cos ^2(\theta )}\partial _\varphi ^2\psi \end{aligned}$$

(see [10, 39]). We briefly write $\Delta _{\mathbb {S}^2}$ as $\Delta$ in this section. At the poles, $\psi$ satisfies $\lim \limits _{\theta \rightarrow \pm {\pi \over 2}}\partial _\varphi \psi (\varphi ,\theta )$ $=0$. Defining

$$\begin{aligned} s=\sin (\theta )\quad \text {and}\quad \psi (\varphi ,\theta )=\Psi (\varphi ,s), \end{aligned}$$

so that

$$\begin{aligned} u=-\sqrt{1-s^2}\,\partial _s \Psi , \qquad v={1\over \sqrt{1-s^2}}\partial _\varphi \Psi , \end{aligned}$$

equation (4.1) can be written as

4.2 $$\begin{aligned} \partial _t\Delta \Psi +\left( -\partial _s\Psi \partial _\varphi +\partial _\varphi \Psi \partial _s\right) (\Delta \Psi +2\omega s)=0, \end{aligned}$$

where

$$\begin{aligned} \Delta \Psi =\partial _s((1-s^2)\partial _s\Psi )+{1\over 1-s^2}\partial _\varphi ^2\Psi , \end{aligned}$$

and $\partial _\varphi \Psi (\varphi ,\pm 1)=0$. Let $\Upsilon =\Delta \Psi$ be the vorticity. Note that $\Psi (\varphi -ct,s)$ is a zonally travelling wave solution to (4.2) if and only if $\Psi (\varphi ,s) + cs$ is a steady solution. Travelling wave solutions of (4.2) can be found by solving the semilinear elliptic problem

4.3 $$\begin{aligned} \Delta \Psi + 2\omega s = G(\Psi + cs) \end{aligned}$$

where *G* is a continuously differentiable function, this being the spherical counterpart of the equation in (3.5). Moreover, throughout regions without critical points of the stream function, any steady solution of (4.2) comes about in this way (see [10, 13]).

### Rigidity of the travelling wave solutions on the rotating sphere

In this subsection, we present two rigidity results. The first regards travelling wave solutions near a zonal flow. The second relies on (4.3) and (4.34), and concerns travelling wave solutions with the minimal zonal period less than $2\pi$.

To study the rigidity of travelling wave solutions near a zonal flow, we first give a characterization of the real spectra of the linearized operator around the zonal flow. This turns out to be quite different from the $\beta$-plane case discussed in Lemma 3.22. For a zonal flow $\Psi _0(s)\in C^3([-1,1])$, the linearized equation around $\Upsilon _0=\Delta \Psi _0$ is

4.4 $$\begin{aligned} \partial _{t}\Upsilon =\Psi _0'\partial _{\varphi }\Upsilon -(\Upsilon _0'+2\omega )\partial _{\varphi }\Delta ^{-1}\Upsilon . \end{aligned}$$

Let $\Upsilon (\varphi ,s)=\sum _{k\ne 0}\Upsilon _k(s)e^{ik\varphi }$ and $\Psi (\varphi ,s)=\sum _{k\ne 0}\Psi _k(s)e^{ik\varphi }$. Decomposing (4.4) into the Fourier modes, we have

$$\begin{aligned} \partial _{t}\Upsilon _k=-ik\mathcal {L}_{k,\omega }\Upsilon _k, \end{aligned}$$

where

4.5 $$\begin{aligned} \mathcal {L}_{k,\omega }\Upsilon _k=-\Psi _{0}'\Upsilon _k+(\Upsilon _0'+2\omega )\Delta _k^{-1}\Upsilon _k \end{aligned}$$

is the linearized operator for the *k*’th mode acting on $L^2(-1,1)$ and

$$\begin{aligned} \Upsilon _k=\Delta _k\Psi _k=((1-s^2)\Psi _k')'-{k^2\over 1-s^2}\Psi _k, \end{aligned}$$

where $k\ne 0$. Since $\mathcal {L}_{k,\omega }$ is a compact perturbation of the multiplication operator $-\Psi _{0}'$, its essential spectra is $\sigma _{ess} (\mathcal {L}_{k,\omega })=Ran(-\Psi _{0}')$. On the other hand, if $\lambda$ is an eigenvalue of $\mathcal {L}_{k,\omega }$, then

$$\begin{aligned}(\Psi _{0}'+\lambda )\Upsilon _k-\Psi _k(\Upsilon _0'+2\omega )=0,\end{aligned}$$

which gives the Rayleigh equation

4.6 $$\begin{aligned} \Delta _k\Psi _k-\frac{\Upsilon _0'+2\omega }{\Psi _0'+\lambda }\Psi _k=0, \quad \Delta _k\Psi _k\in L^2(-1,1). \end{aligned}$$

We call $(\lambda ,k,\omega ,\Psi )$ with $\lambda \in \mathbb {R}$ a neutral mode if $\Psi$ is a non-trivial solution of (4.6). We characterize now the real spectra of the linearized operator $\mathcal {L}_{k,\omega }$.

#### Lemma 4.1

Let $\Psi _0\in C^3([-1,1])$ and $k\ne 0$. Then

(i) if $$-\omega \in Ran(-\Psi _{0}')$$, then $$\sigma (\mathcal {L}_{k,\omega })\cap \mathbb {R}=Ran(-\Psi _{0}')$$;

(ii) if $(-\Psi _{0}')_{\max }<-\omega$, then $\sigma (\mathcal {L}_{k,\omega })\cap \mathbb {R}\subset [(-\Psi _{0}')_{\min },-\omega ]$;

(iii) if $(-\Psi _{0}')_{\min }>-\omega$, then $\sigma (\mathcal {L}_{k,\omega })\cap \mathbb {R}\subset [-\omega ,(-\Psi _{0}')_{\max }]$.

In terms of the neutral modes, Lemma 4.1 can be restated as follows.

**Restatement of Lemma** 4.1. Let $\Psi _0\in C^3([-1,1])$ and $k\ne 0$. Then

(i) if $-\omega \in Ran(-\Psi _{0}')$, then for any neutral mode $(\lambda ,k,\omega ,\Psi )$, $\lambda$ must lie in $Ran(-\Psi _{0}')$;

(ii) if $(-\Psi _{0}')_{\max }<-\omega$, then for any neutral mode $(\lambda ,k,\omega ,\Psi )$, $\lambda$ must lie in $[(-\Psi _{0}')_{\min },-\omega ]$;

(iii) if $(-\Psi _{0}')_{\min }>-\omega$, then for any neutral mode $(\lambda ,k,\omega ,\Psi )$, $\lambda$ must lie in $[-\omega ,(-\Psi _{0}')_{\max }]$.

#### Proof

Let $\lambda \notin Ran(-\Psi _{0}')$. For $s\in [-1,1]$, we define

$$\begin{aligned}R(s)=\Psi _{0}'(s)+\lambda ,\;F(s)=\frac{\Psi (s)}{R(s)}=\frac{\Psi (s)}{\Psi _{0}'(s)+\lambda }.\end{aligned}$$

If $\Psi$ solves (4.6), then we have

$$\begin{aligned}-R\left[ \left( \left( 1-s^2\right) (RF)'\right) '-\frac{k^2RF}{1-s^2}\right] +RF\left[ \left( 1-s^2\right) (R-\lambda )\right] ''+2\omega RF=0.\end{aligned}$$

From the identity

$$\begin{aligned}  &   RF\left( \left( 1-s^2\right) R\right) ''-R\left( \left( 1-s^2\right) (RF)'\right) '+\frac{1}{\sqrt{1-s^2}}\left( \left( \frac{F}{\sqrt{1-s^2}}\right) '(1-s^2)^2R^2\right) '\\  &   \quad =-\frac{FR^2}{1-s^2},\end{aligned}$$

it follows that

4.7 $$\begin{aligned} -\frac{1}{\sqrt{1-s^2}}\left( \left( \frac{F}{\sqrt{1-s^2}}\right) '(1-s^2)^2R^2\right) '+\frac{FR^2}{1-s^2}(k^2-1)+2RF(\lambda +\omega )=0,\nonumber \\ \end{aligned}$$

with $F(\pm 1)=0$. Multiplying (4.7) by *F*, integrating over $[-1,1]$ and performing integration by parts give

$$\begin{aligned}\int _{-1}^{1}\left| \left( \frac{F}{\sqrt{1-s^2}}\right) '\right| ^2(1-s^2)^2R^2ds +\int _{-1}^{1}\frac{F^2R^2}{1-s^2}(k^2-1)ds+\int _{-1}^{1}2RF^2(\lambda +\omega )ds=0.\end{aligned}$$

Thus

$$\begin{aligned}\int _{-1}^{1}R^2\left( \left| \left( \frac{F}{\sqrt{1-s^2}}\right) '\right| ^2(1-s^2)^2 +\frac{F^2}{1-s^2}(k^2-1)\right) ds+\int _{-1}^{1}2RF^2(\lambda +\omega )ds=0.\end{aligned}$$

Denoting

$$\begin{aligned}P(s)=\left| \left( \frac{F}{\sqrt{1-s^2}}\right) '\right| ^2(1-s^2)^2+\frac{(k^2-1)F^2}{1-s^2}\ge 0,\quad k\ne 0,\end{aligned}$$

we obtain

4.8 $$\begin{aligned} \int _{-1}^{1}PR^2ds+\int _{-1}^{1}2RF^2(\lambda +\omega )ds=0. \end{aligned}$$

First, we prove part (i) of the statement. Suppose that $\lambda \notin Ran(-\Psi _{0}')$. If $\lambda <(-\Psi _{0}')_{\min }$, then $\lambda <-\omega$. On the other hand, we have $R(s)=\Psi _{0}'(s)+\lambda <0$ for $s\in [-1,1]$. Thus $R(s)(\lambda +\omega )>0$ for $s\in [-1,1]$ and (4.8) yields $F=0$. But then $\Psi =0$, which is a contradiction.

If $\lambda >(-\Psi _{0}')_{\max }$, then $\lambda >-\omega$ and $R(s)=\Psi _{0}'(s)+\lambda >0$ for $s\in [-1,1]$. This implies $R(s)(\lambda +\omega )>0$ for $s\in [-1,1]$. By (4.8) we get $F=0$ and thus $\Psi =0$, which is also a contradiction. We must therefore have $\lambda \in Ran(-\Psi _{0}')$.

We now prove part (ii) of the statement. Suppose that $\lambda \notin [(-\Psi _{0}')_{\min },-\omega ]$. If $\lambda <(-\Psi _{0}')_{\min }$, then $\lambda <-\omega$ and $R(s)<0$ for $s\in [-1,1]$. Then $R(s)(\lambda +\omega )>0$ for $s\in [-1,1]$ and (4.8) yields $F=0$. Thus $\Psi =0$, which is a contradiction.

If $$\lambda >-\omega $$, then

$$\begin{aligned}(-\Psi _{0}')_{\max }<-\omega <\lambda ,\end{aligned}$$

and we get $R(s)>0$ for $s\in [-1,1]$. This yields $R(s)(\lambda +\omega )>0$ for $s\in [-1,1]$. From (4.8) we get $F=0$ and thus $\Psi =0$, which is also a contradiction. Consequently $\lambda \in [(-\Psi _{0}')_{\min },-\omega ]$.

Finally, we prove part (iii) of the statement. Suppose that $\lambda \notin [-\omega ,(-\Psi _{0}')_{\max }]$. If $\lambda <-\omega$, then

$$\begin{aligned}\lambda<-\omega <(-\Psi _{0}')_{\min },\end{aligned}$$

and $R(s)<0$ for $s\in [-1,1]$. Then $R(s)(\lambda +\omega )>0$ for $s\in [-1,1]$. From (4.8) we obtain $F=0$ and thus $\Psi =0$, which is a contradiction.

If $\lambda >(-\Psi _{0}')_{\max }$, then $\lambda >-\omega$ and $R(s)>0$ for $s\in [-1,1]$. This yields $R(s)(\lambda +\omega )>0$ for $s\in [-1,1]$. From (4.8) we get $F=0$ and thus $\Psi =0$, which is also a contradiction. Therefore $\lambda \in [-\omega ,(-\Psi _{0}')_{\max }]$. $\square$

We now introduce the Sobolev spaces on the unit sphere $\mathbb {S}^2=\{\textbf{x}=(x,y,z)\in \mathbb {R}^3|x^2+y^2+z^2=1\}$, based on the theory of Sobolev spaces on general compact Riemannian manifolds [4, 23]. The Riemannian metric *g* of $\mathbb {S}^2$ is induced by the Euclidean metric of $\mathbb {R}^3$. For $f\in C^\infty (\mathbb {S}^2)$, set $\Vert \nabla ^k f\Vert _{L^p(\mathbb {S}^2)}= \bigg (\int _{\mathbb {S}^2}|\nabla ^kf|^pd\sigma _g\bigg )^{1\over p}$ and $\Vert f\Vert _{H_k^p(\mathbb {S}^2)}=\sum \limits _{j=0}^k\Vert \nabla ^j f\Vert _{L^p(\mathbb {S}^2)}$ for $p\ge 1$ and $k\ge 0$, where $\nabla ^k f$ denotes the *k*-th covariant derivative of *f* and $|\nabla ^k f|$ is defined in Definition 2.2 of [4]. The Sobolev space $H_{k}^p(\mathbb {S}^2)$ with $k \ge 0$ is defined as the completion of $C^{\infty }(\mathbb {S}^2)$ with respect to $\Vert \cdot \Vert _{H_k^p}$. Further, for any integer $m \ge 0$ we endow $C^m(\mathbb {S}^2)$ with the norm $\Vert f\Vert _{C^m(\mathbb {S}^2)}=\sum \limits _{j=0}^m\max _{\textbf{x}\in \mathbb {S}^2}|(\nabla ^j f)(\textbf{x})|$.

With the help of Lemma 4.1, we are now in a position to present a rigidity result for travelling wave solutions near a zonal flow on a rotating sphere. In the following theorem and its proof, we identify a point $\textbf{x}\in \mathbb {S}^2$ and its geographic coordinates $(\varphi ,s)$.

#### Theorem 4.2

Let $\omega \ge 0$, $p\ge 3$ and $\delta >0$. Suppose that $\Psi _0 \in C^3([-1,1])$ is a zonal flow on the unit sphere rotating with speed $\omega$. Then there exists $\epsilon _{\delta }>0$ such that any travelling wave solution $\Psi (\varphi -ct,s)$ to the Euler equation (4.2) satisfying

4.9 $$\begin{aligned} \left\Vert \Upsilon -\Upsilon _0\right\Vert _{H_{p}^{2}(\mathbb {S}^2)} +\left\Vert \partial _{s}\Psi -\Psi _{0}'\right\Vert _{C(\mathbb {S}^2)}\le \epsilon _{\delta }, \end{aligned}$$

where $\Upsilon =\Delta \Psi$ and $\Upsilon _0=\Delta \Psi _0\in H_{p}^{2}(\mathbb {S}^2)$, and such that the wave speed *c* satisfies one of the following conditions,

(i) $$c\notin I_{\delta }\triangleq \left( (-\Psi _{0}')_{\min }-\delta ,(-\Psi _{0}')_{\max }+\delta \right) $$ if $$-\omega \in Ran(-\Psi _{0}')$$,

(ii) $$c\notin I_{\delta }\triangleq ((-\Psi _{0}')_{\min }-\delta ,-\omega +\delta )$$ if $$-\omega >(-\Psi _{0}')_{\max }$$,

(iii) $$c\notin I_{\delta }\triangleq (-\omega -\delta ,(-\Psi _{0}')_{\max }+\delta )$$ if $$-\omega <(-\Psi _{0}')_{\min }$$,

then $\Psi (\varphi -ct,s)$ must be a zonal flow.

#### Proof of of Theorem 4.2

Since $\Psi _0 \in C^3([-1,1])$, $Ran(-\Psi _{0}')$ and $Ran(\Upsilon _0')=Ran\big (((1-s^2)\Psi _0')''\big )$ must be compact and connected, and thus is a compact interval.

Suppose that for any $n>0$, there exists a travelling wave solution $\Psi _n(\varphi -c_nt,s)$ to the Euler equation (4.2) satisfying

4.10 $$\begin{aligned} \left\Vert \Upsilon _n-\Upsilon _0\right\Vert _{H_{p}^{2}(\mathbb {S}^2)} +\left\Vert \partial _{s}\Psi _n-\Psi _{0}'\right\Vert _{C(\mathbb {S}^2)}\le \frac{1}{n}, \end{aligned}$$

and $c_n\notin I_\delta$, but $\partial _{\varphi }\Psi _n\not \equiv 0$, where $\Upsilon _n=\Delta \Psi _n$. By (4.2), $\Psi _n$ solves

4.11 $$\begin{aligned} -(c_n+\partial _{s}\Psi _n)\partial _{\varphi }\Delta \Psi _n+\partial _{\varphi }\Psi _n(\partial _{s}\Delta \Psi _n+2\omega )=0. \end{aligned}$$

Let *n* be large enough so that $1/n<\delta /2$. Then for $s\in [-1,1]$,

4.12 $$\begin{aligned} |\partial _{s}\Psi _n+c_n| \ge |\Psi _0'+c_n|-|\partial _{s}\Psi _n-\Psi _0'|\ge \delta -\frac{1}{n}>\frac{\delta }{2}. \end{aligned}$$

Thus, by (4.11) we have

4.13 $$\begin{aligned} -\partial _{\varphi }\Delta \Psi _n+\frac{\partial _{\varphi }\Psi _n(\partial _{s}\Delta \Psi _n+2\omega )}{c_n+\partial _{s}\Psi _n}=0. \end{aligned}$$

Note that

$$\begin{aligned}0\ne \left\Vert \partial _{\varphi }\Psi _n\right\Vert _{L^{2}(\mathbb {S}^2)} \le \left\Vert \frac{1}{\sqrt{1-s^2}}\partial _{\varphi }\Psi _n\right\Vert _{L^{2}(\mathbb {S}^2)} \le \left\Vert \Psi _n\right\Vert _{H^{2}_{1}(\mathbb {S}^2)}\le C\end{aligned}$$

for $n\ge 1$. We normalize $\partial _{\varphi }\Psi _n$ by $\xi _n=\frac{\partial _{\varphi }\Psi _n}{\left\Vert \partial _{\varphi }\Psi _n\right\Vert _{L^{2}(\mathbb {S}^2)}}$ such that $\left\Vert \xi _n\right\Vert _{L^{2}(\mathbb {S}^2)}=1$. It follows from (4.13) that

4.14 $$\begin{aligned} -\Delta \xi _n+\xi _n\frac{\partial _{s}\Delta \Psi _n+2\omega }{c_n+\partial _{s}\Psi _n}=0. \end{aligned}$$

By Theorem 2.7 in [23], $H_{3}^{2}(\mathbb {S}^2)$ is embedded in $C^{1}(\mathbb {S}^2)$. So

$$\begin{aligned} \begin{aligned} \left\Vert \Delta \Psi _n+2\omega s\right\Vert _{C^{1}(\mathbb {S}^2)}&\le \left\Vert \Delta \Psi _n\right\Vert _{C^{1}(\mathbb {S}^2)}+C \le \left\Vert \Delta \Psi _n-\Delta \Psi _0\right\Vert _{C^{1}(\mathbb {S}^2)}+\left\Vert \Delta \Psi _0\right\Vert _{C^{1}(\mathbb {S}^2)}+C\\&\le C\left\Vert \Delta \Psi _n-\Delta \Psi _0\right\Vert _{H_{3}^{2}(\mathbb {S}^2)}+C\left\Vert \Delta \Psi _0\right\Vert _{H_{3}^{2}(\mathbb {S}^2)}+C\\&\le \frac{C}{n}+C\le C \end{aligned} \end{aligned}$$

for $n\ge 1$. This, together with (4.12) and (4.14), yields

4.15 $$\begin{aligned} \begin{aligned} \left\Vert \Delta \xi _n\right\Vert _{L^{2}(\mathbb {S}^2)}&=\left\Vert \xi _n\frac{\partial _{s}(\Delta \Psi _n+2\omega s)}{c_n+\partial _{s}\Psi _n}\right\Vert _{L^{2}(\mathbb {S}^2)}\\&\le \frac{2}{\delta }\left\Vert \frac{\xi _n}{\sqrt{1-s^2}}\sqrt{1-s^2}\partial _{s}(\Delta \Psi _n +2\omega s)\right\Vert _{L^{2}(\mathbb {S}^2)}\\&\le \frac{2C}{\delta }\left\Vert \frac{\xi _n}{\sqrt{1-s^2}}\right\Vert _{L^{2}(\mathbb {S}^2)}. \end{aligned} \end{aligned}$$

By the definition of $\xi _n$, its zero’th Fourier mode is 0 and thus, we write $\xi _n(\varphi ,s)=\sum \limits _{k\ne 0}e^{ik\varphi }\xi _{n,k}(s)$. Then

4.16 $$\begin{aligned} \begin{aligned}&\left\Vert \frac{\xi _n}{\sqrt{1-s^2}}\right\Vert _{L^{2}(\mathbb {S}^2)}^{2} =\int _{\mathbb {S}^2}\frac{|\xi _n|^2}{1-s^2}d\sigma _g\\ =&\int _{0}^{2\pi }\int _{-1}^{1}\frac{1}{1-s^2}\sum _{k_1\ne 0}e^{ik_1\varphi }\xi _{n,k_1}(s) \sum _{k_2\ne 0}e^{-ik_2\varphi }\overline{\xi _{n,k_2}(s)}dsd\varphi \\ =&\int _{-1}^{1}\frac{1}{1-s^2}\int _{0}^{2\pi }\sum _{k\ne 0}|\xi _{n,k}(s)|^2d\varphi ds \le \int _{-1}^{1}\frac{1}{1-s^2}\int _{0}^{2\pi }\sum _{k\ne 0}k^2|\xi _{n,k}(s)|^2d\varphi ds\\ =&\int _{0}^{2\pi }\int _{-1}^{1}\frac{1}{1-s^2}\sum _{k_1\ne 0}ik_1e^{ik_1\varphi }\xi _{n,k_1}(s) \sum _{k_2\ne 0}-ik_2e^{-ik_2\varphi }\overline{\xi _{n,k_2}(s)}dsd\varphi \\ =&\int _{0}^{2\pi }\int _{-1}^{1}\frac{1}{1-s^2}\partial _{\varphi }\xi _n\overline{\partial _{\varphi }\xi _n}dsd\varphi =\int _{\mathbb {S}^2}\frac{|\partial _{\varphi }\xi _n|^2}{1-s^2}d\sigma _g =\left\Vert \frac{\partial _{\varphi }\xi _n}{\sqrt{1-s^2}}\right\Vert _{L^{2}(\mathbb {S}^2)}^{2}. \end{aligned}\nonumber \\ \end{aligned}$$

By (4.15) and (4.16) we have

$$\begin{aligned} \begin{aligned} \left\Vert \Delta \xi _n\right\Vert _{L^{2}(\mathbb {S}^2)}&\le \frac{2C}{\delta }\left\Vert \frac{\partial _{\varphi }\xi _n}{\sqrt{1-s^2}}\right\Vert _{L^{2}(\mathbb {S}^2)} \le \frac{2C}{\delta }\left\Vert \nabla \xi _n\right\Vert _{L^{2}(\mathbb {S}^2)} \le \frac{2C}{\delta }\left\Vert \xi _n\right\Vert _{H_{1}^{2}(\mathbb {S}^2)}\\&\le \frac{2C}{\delta }\left\Vert \xi _n\right\Vert _{L^{2}(\mathbb {S}^2)}^{\frac{1}{2}} \left\Vert \xi _n\right\Vert _{H_{2}^{2}(\mathbb {S}^2)}^{\frac{1}{2}}. \end{aligned} \end{aligned}$$

This, together with the fact that $\left\Vert \xi _n\right\Vert _{H_{2}^{2}(\mathbb {S}^2)}\le C\left\Vert \Delta \xi _n\right\Vert _{L^{2}(\mathbb {S}^2)}$, yields

$$\begin{aligned}\left\Vert \xi _n\right\Vert _{H_{2}^{2}(\mathbb {S}^2)}\le \frac{2C}{\delta }\left\Vert \xi _n\right\Vert _{L^{2}(\mathbb {S}^2)}^{\frac{1}{2}} \left\Vert \xi _n\right\Vert _{H_{2}^{2}(\mathbb {S}^2)}^{\frac{1}{2}}.\end{aligned}$$

Then

4.17 $$\begin{aligned} \left\Vert \xi _n\right\Vert _{H_{2}^{2}(\mathbb {S}^2)}^{\frac{1}{2}} \le \frac{2C}{\delta }\left\Vert \xi _n\right\Vert _{L^{2}(\mathbb {S}^2)}^{\frac{1}{2}}=\frac{2C}{\delta }. \end{aligned}$$

Thus, $\{\xi _n\}_{n\ge 1}$ is uniformly bounded in $H_{2}^{2}(\mathbb {S}^2)$. Consequently there exists $\xi _0\in H_{2}^{2}(\mathbb {S}^2)$ such that $\xi _n\rightharpoonup \xi _0$ in $H_{2}^{2}(\mathbb {S}^2)$, $\xi _n\rightarrow \xi _0$ in $H_{1}^{2}(\mathbb {S}^2)$ and $\left\Vert \xi _0\right\Vert _{L^{2}(\mathbb {S}^2)}=1$. For any $\Phi \in H_{1}^{2}(\mathbb {S}^2)$, by (4.14), we have

4.18 $$\begin{aligned} \int _{\mathbb {S}^2}\left( \nabla \xi _n\cdot \nabla \overline{\Phi }+\xi _n\overline{\Phi }\frac{\partial _{s}\Delta \Psi _n-\Delta \Psi _0'}{c_n+\partial _{s}\Psi _n} +\xi _n\overline{\Phi }\frac{\Delta \Psi _0'+2\omega }{c_n+\partial _{s}\Psi _n}\right) d\sigma _g=0. \end{aligned}$$

For *n* large enough such that $1/n<\delta /2$, by (4.12) we have

4.19 $$\begin{aligned} \begin{aligned} \left| \int _{\mathbb {S}^2}\xi _n\overline{\Phi }\frac{\partial _{s}\Delta \Psi _n-\Delta \Psi _0'}{c_n+\partial _{s}\Psi _n}d\sigma _g\right|&\le \frac{2}{\delta }\left| \int _{\mathbb {S}^2}\xi _n\overline{\Phi }(\partial _{s}\Delta \Psi _n-\Delta \Psi _0')d\sigma _g\right| \\&=\frac{2}{\delta }\left| \int _{\mathbb {S}^2}\frac{\xi _n}{\sqrt{1-s^2}}\overline{\Phi }\sqrt{1-s^2}\partial _{s}(\Delta \Psi _n-\Delta \Psi _0)d\sigma _g\right| . \end{aligned} \end{aligned}$$

Since $H_{3}^{2}(\mathbb {S}^2)$ is embedded in $C^{1}(\mathbb {S}^2)$, by (4.10) we have

$$\begin{aligned} \left| \sqrt{1-s^2}\partial _{s}(\Delta \Psi _n-\Delta \Psi _0)\right|  &   \le \left\Vert \Delta \Psi _n-\Delta \Psi _0\right\Vert _{C^{1}(\mathbb {S}^2)}\\  &   \le C\left\Vert \Delta \Psi _n-\Delta \Psi _0\right\Vert _{H_{3}^{2}(\mathbb {S}^2)} \le \frac{C}{n}\rightarrow 0 \end{aligned}$$

as $n\rightarrow +\infty$ uniformly for $(\varphi ,s)\in \mathbb {T}_{2\pi }\times [-1,1]$. This, together with (4.16)-(4.17) and (4.19), implies that

4.20 $$\begin{aligned} \begin{aligned} \left| \int _{\mathbb {S}^2}\xi _n\overline{\Phi }\frac{\partial _{s}\Delta \Psi _n-\Delta \Psi _0'}{c_n+\partial _{s}\Psi _n}d\sigma _g\right| \le&\frac{2}{\delta }\frac{C}{n}\left\Vert \frac{\xi _n}{\sqrt{1-s^2}}\right\Vert _{L^{2}(\mathbb {S}^2)} \left\Vert \Phi \right\Vert _{L^{2}(\mathbb {S}^2)}\\ \le&\frac{2}{\delta }\frac{C}{n}\left\Vert \frac{\partial _{\varphi }\xi _n}{\sqrt{1-s^2}}\right\Vert _{L^{2}(\mathbb {S}^2)} \left\Vert \Phi \right\Vert _{L^{2}(\mathbb {S}^2)}\\ \le&\frac{2}{\delta }\frac{C}{n}\left\Vert \xi _n\right\Vert _{H_{1}^{2}(\mathbb {S}^2)} \left\Vert \Phi \right\Vert _{L^{2}(\mathbb {S}^2)} \le \frac{2}{\delta ^3}\frac{C}{n}\left\Vert \Phi \right\Vert _{L^{2}(\mathbb {S}^2)}\rightarrow 0 \end{aligned} \end{aligned}$$

as $n\rightarrow +\infty$. To estimate the last term in (4.18), we divide the proof into two cases.

**Case 1**. Up to a subsequence, $c_n\rightarrow +\infty$.

Due to the fact that $Ran(\Psi _0')$ is compact, by (4.10) we have

$$\begin{aligned}|\partial _{s}\Psi _n|\le |\partial _{s}\Psi _n-\Psi _0'|+|\Psi _0'|\le \frac{1}{n}+C\le C\end{aligned}$$

for $n\ge 1$ and $(\varphi ,s)\in \mathbb {T}_{2\pi }\times [-1,1]$. Then

4.21 $$\begin{aligned} |c_n+\partial _{s}\Psi _n|\ge |c_n|-|\partial _{s}\Psi _n|\ge |c_n|-C \end{aligned}$$

for any $(\varphi ,s)\in \mathbb {T}_{2\pi }\times [-1,1]$ and $n\ge 1$. Moreover,

4.22 $$\begin{aligned} \sqrt{1-s^2}\left| \Delta \Psi _0'+2\omega \right| \le \left\Vert \Delta \Psi _0\right\Vert _{C^{1}(\mathbb {S}^2)}+C \le C\left\Vert \Delta \Psi _0\right\Vert _{H_{3}^{2}(\mathbb {S}^2)}+C\le C \end{aligned}$$

for any $(\varphi ,s)\in \mathbb {T}_{2\pi }\times [-1,1]$. For *n* large enough, it follows from (4.16), (4.17), (4.21) and (4.22) that

4.23 $$\begin{aligned} \begin{aligned} \left| \int _{\mathbb {S}^2}\xi _n\overline{\Phi }\frac{\Delta \Psi _0'+2\omega }{c_n+\partial _{s}\Psi _n}d\sigma _g\right|&\le \frac{1}{|c_n|-C}\int _{\mathbb {S}^2}\left| \frac{\xi _n}{\sqrt{1-s^2}}\overline{\Phi }\sqrt{1-s^2}(\Delta \Psi _0'+2\omega )\right| d\sigma _g\\&\le \frac{C}{|c_n|-C}\left\Vert \frac{\xi _n}{\sqrt{1-s^2}}\right\Vert _{L^{2}(\mathbb {S}^2)}\left\Vert \Phi \right\Vert _{L^{2}(\mathbb {S}^2)}\\&\le \frac{C}{|c_n|-C}\left\Vert \frac{\partial _{\varphi }\xi _n}{\sqrt{1-s^2}}\right\Vert _{L^{2}(\mathbb {S}^2)} \left\Vert \Phi \right\Vert _{L^{2}(\mathbb {S}^2)}\\&\le \frac{C}{|c_n|-C}\left\Vert \xi _n\right\Vert _{H_{1}^{2}(\mathbb {S}^2)}\left\Vert \Phi \right\Vert _{L^{2}(\mathbb {S}^2)} \le \frac{C}{(|c_n|-C)\delta ^2}\left\Vert \Phi \right\Vert _{L^{2}(\mathbb {S}^2)}\\&\rightarrow 0 \end{aligned} \end{aligned}$$

as $n\rightarrow +\infty$. Taking $n\rightarrow +\infty$ in (4.18), by (4.20), (4.23) and the fact that $\xi _n\rightarrow \xi _0$ in $H_{1}^{2}(\mathbb {S}^2)$ we have

$$\begin{aligned}\int _{\mathbb {S}^2}\nabla \xi _0\cdot \nabla \overline{\Phi } d\sigma _g=0.\end{aligned}$$

Choose $\Phi =\xi _0\in H_1^2(\mathbb {S}^2)$. Then $\xi _0=0$, which contradicts $\left\Vert \xi _0\right\Vert _{L^{2}(\mathbb {S}^2)}=1$.

**Case 2.** Up to a subsequence, $c_n\rightarrow c_0\in \mathbb {R}$.

Without loss of generality, suppose that $c_0$ lies on the left hand side of $I_{\delta }$. By (4.12) and $c_n\notin I_\delta$, we have

$$\begin{aligned} \begin{aligned}&\left| \int _{\mathbb {S}^2}\left( \xi _n\overline{\Phi }\frac{\Delta \Psi _0'+2\omega }{c_n+\partial _{s}\Psi _n} -\xi _0\overline{\Phi }\frac{\Delta \Psi _0'+2\omega }{c_0+\Psi _0'}\right) d\sigma _g\right| \\ \le&\int _{\mathbb {S}^2}\left| \overline{\Phi }(\Delta \Psi _0'+2\omega )\frac{\xi _n(c_0+\Psi _0')-\xi _0(c_n+\partial _{s}\Psi _n)}{(c_n+\partial _{s}\Psi _n)(c_0+\Psi _0')}\right| d\sigma _g\\ \le&\frac{2}{\delta ^2}\int _{\mathbb {S}^2}|\overline{\Phi }(\Delta \Psi _0'+2\omega )\xi _n(c_0-c_n)|d\sigma _g +\frac{2}{\delta ^2}\int _{\mathbb {S}^2}|\overline{\Phi }(\Delta \Psi _0'+2\omega )c_n(\xi _n-\xi _0)|d\sigma _g\\&+\frac{2}{\delta ^2}\int _{\mathbb {S}^2}|\overline{\Phi }(\Delta \Psi _0'+2\omega )\Psi _0'(\xi _n-\xi _0)|d\sigma _g +\frac{2}{\delta ^2}\int _{\mathbb {S}^2}|\overline{\Phi }(\Delta \Psi _0'+2\omega )\xi _0(\Psi _0'-\partial _{s}\Psi _n)|d\sigma _g\\ \le&\frac{2}{\delta ^2}\left\Vert \Phi \right\Vert _{L^{2}(\mathbb {S}^2)}\left( {C\over \delta ^2}|c_n-c_0|+C\left\Vert \xi _n-\xi _0\right\Vert _{H_{1}^{2}(\mathbb {S}^2)} +C\left\Vert \xi _n-\xi _0\right\Vert _{H_{1}^{2}(\mathbb {S}^2)} +{C\over \delta ^2}\left\Vert \partial _{s}\Psi _n-\Psi _0'\right\Vert _{C(\mathbb {S}^2)}\right) \end{aligned} \end{aligned}$$

for *n* large enough. This implies that

4.24 $$\begin{aligned} \int _{\mathbb {S}^2}\xi _n\overline{\Phi }\frac{\Delta \Psi _0'+2\omega }{c_n+\partial _{s}\Psi _n}d\sigma _g \rightarrow \int _{\mathbb {S}^2}\xi _0\overline{\Phi }\frac{\Delta \Psi _0'+2\omega }{c_0+\Psi _0'}d\sigma _g \end{aligned}$$

as $n\rightarrow +\infty$. Thus, taking $n\rightarrow +\infty$ in (4.18), by (4.20), (4.24) and the fact that $\xi _n\rightarrow \xi _0$ in $H_{1}^{2}(\mathbb {S}^2)$ we have

4.25 $$\begin{aligned} \int _{\mathbb {S}^2}\left( \nabla \xi _0\cdot \nabla \overline{\Phi } +\xi _0\overline{\Phi }\frac{\Delta \Psi _0'+2\omega }{c_0+\Psi _0'}\right) d\sigma _g=0. \end{aligned}$$

Since $\xi _0(\varphi ,s)=\sum \limits _{k\in \mathbb {Z}}e^{ik\varphi }\xi _{0,k}(s)\ne 0$, there exists $k_0\in \mathbb {Z}$ such that $\xi _{0,k_0}\ne 0$.

We claim that $k_0\ne 0$. In fact, for $n\ge 1$,

$$\begin{aligned}\xi _{n,0}=\frac{1}{2\pi }\int _{0}^{2\pi }\xi _nd\varphi =\frac{1}{2\pi }\int _{0}^{2\pi }\frac{\partial _{\varphi }\Psi _n}{\left\Vert \partial _{\varphi }\Psi _n\right\Vert _{L^{2}(\mathbb {S}^2)}}d\varphi =0,\end{aligned}$$

and

$$\begin{aligned}\left\Vert \xi _{n,0}-\xi _{0,0}\right\Vert _{L^{2}(\mathbb {S}^2)}^2  &   =\int _{\mathbb {S}^2}|\xi _{n,0}-\xi _{0,0}|^2d\sigma _g \le \sum _{k\in \mathbb {Z}}\int _{\mathbb {S}^2}|\xi _{n,k}-\xi _{0,k}|^2d\sigma _g\\  &   =\left\Vert \xi _{n}-\xi _{0}\right\Vert _{L^{2}(\mathbb {S}^2)}^{2}\rightarrow 0\end{aligned}$$

as $$n\rightarrow +\infty $$. Thus,

$$\begin{aligned}\left\Vert \xi _{0,0}\right\Vert _{L^{2}(\mathbb {S}^2)} \le \left\Vert \xi _{n,0}-\xi _{0,0}\right\Vert _{L^{2}(\mathbb {S}^2)} +\left\Vert \xi _{n,0}\right\Vert _{L^{2}(\mathbb {S}^2)}\rightarrow 0\end{aligned}$$

as $n\rightarrow +\infty$. This implies that $\xi _{0,0}=0$ and hence $k_0\ne 0$.

Inserting $\Phi (\varphi ,s)=e^{ik_0\varphi }\xi _{0,k_0}(s)$ into (4.25), we get

$$\begin{aligned} \int _{\mathbb {S}^2}\left( \nabla (\xi _{0,k_0}e^{ik_0\varphi })\cdot \nabla (\xi _{0,-k_0}e^{-ik_0\varphi }) +\xi _{0,k_0}e^{ik_0\varphi }\xi _{0,-k_0}e^{-ik_0\varphi }\frac{\Delta \Psi _0'+2\omega }{c_0+\Psi _0'}\right) d\sigma _g=0. \end{aligned}$$

Then

4.26 $$\begin{aligned} \int _{-1}^{1}\left( |\nabla _{k_0}\xi _{0,k_0}|^2+|\xi _{0,k_0}|^2\frac{\Delta \Psi _0'+2\omega }{c_0+\Psi _0'}\right) ds=0 \end{aligned}$$

and $$\nabla _{k_0}\xi _{0,k_0}\in L^2(-1,1)$$.

Denote now the left endpoint of $I_{\delta }$ by $a_{\delta }$ and consider the eigenvalue problem

4.27 $$\begin{aligned} -\Delta _{k_0}\Phi +\frac{\Delta \Psi _0'+2\omega }{c+\Psi _0'}\Phi =\lambda \Phi ,\quad \Delta _{k_0}\Phi \in L^2(-1,1), \end{aligned}$$

where $c\in (-\infty ,a_{\delta }]$. Since $Ran(\Delta \Psi _0')$ is compact, the principal (i.e., minimal) eigenvalue of (4.27) is

4.28 $$\begin{aligned} \lambda _1(c)=\inf _{\nabla _{k_0}\Phi \in L^{2}(-1,1)}\frac{\int _{-1}^{1}\left( |\nabla _{k_0}\Phi |^2+\frac{\Delta \Psi _0'+2\omega }{c+\Psi _0'}|\Phi |^2\right) ds}{\int _{-1}^{1}|\Phi |^2ds}. \end{aligned}$$

Here, we note that $\Vert \Phi \Vert _{L^2(-1,1)}\le \Vert \nabla _{k_0}\Phi \Vert _{L^2(-1,1)}$ by the Poincaré inequality.

We claim that $\lambda _1(c)>0$ for any $c\in (-\infty ,a_{\delta }]$. First, we note that $\frac{\Delta \Psi _0'+2\omega }{c+\Psi _0'}\rightarrow 0$ as $c\rightarrow -\infty$ uniformly for $s\in [-1,1]$, and thus,

$$\begin{aligned}\lim _{c\rightarrow -\infty }\lambda _1(c)=k_0(k_0+1).\end{aligned}$$

Fix $\hat{c}\in (-\infty ,a_{\delta }]$. By Lemma 4.1, $\lambda _1(\hat{c})\ne 0$. If $\lambda _1(\hat{c})<0$, then there exists $c_{*}\in (-\infty ,\hat{c}]$ such that $\lambda _1(c_{*})=0$. Then $(c_{*},k,\omega ,\Phi _{*})$ is a neutral mode, where $\Phi _{*}$ is an eigenfunction of $\lambda _1(c_{*})$, which contradicts Lemma 4.1. Thus, $\lambda _1(\hat{c})>0$.

By (4.26), (4.28) and $c_0\in (-\infty ,a_{\delta }]$, we have

$$\begin{aligned}\lambda _1(c_0)\le \frac{\int _{-1}^{1}\left( |\nabla _{k_0}\xi _{0,k_0}|^2 +\frac{\Delta \Psi _0'+2\omega }{c_0+\Psi _0'}|\xi _{0,k_0}|^2\right) ds}{\int _{-1}^{1}|\xi _{0,k_0}|^2ds}=0,\end{aligned}$$

which contradicts $$\lambda _1(c_0)>0$$. $$\square $$

#### Remark 4.3

There are some fundamental differences with respect to the rigidity of zonally shear flows between the spherical geometry and the flat-space geometry of the $\beta$-plane.

(i) Let us first discuss the difference between the real spectra of the linearized operator $\mathbb {L}_{k\alpha ,\beta }$ (defined in (3.42)) around a shear flow $\psi _{0}(y)$ in the flat-space geometry and the real spectra of the linearized operator $\mathcal {L}_{k,\omega }$ (defined in (4.5)) around a zonal flow $\Psi _0(s)$ in the spherical geometry.

**Fig. 7:**
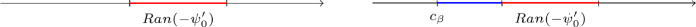
The real spectra of the linearized operator $\mathbb {L}_{k\alpha ,\beta }$ are exactly $Ran(-\psi _{0}')$ for $\beta =0$ (left) and are larger than $Ran(-\psi _{0}')$ from the left hand side for $\beta >0$ in general (right). The isolated real eigenvalues may lie in $[c_\beta ,(-\psi _0')_{\min })$ for $\beta >0$

In the flat-space geometry, by Lemma 3.22 whether $\beta =0$ or not affects the real spectra of the linearized operator $\mathbb {L}_{k\alpha ,\beta }$, as shown in Fig. 7.

**Fig. 8:**
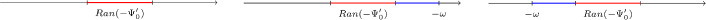
The real spectra of the linearized operator $\mathcal {L}_{k,\omega }$ are exactly $Ran(-\Psi _{0}')$ for $-\omega \in Ran(-\Psi _{0}')$ (left), are larger than $Ran(-\Psi _{0}')$ from the right hand side for $(-\Psi _{0}')_{\max }<-\omega$ in general (middle), and are larger than $Ran(-\Psi _{0}')$ from the left hand side for $(-\Psi _{0}')_{\min }>-\omega$ in general (right)

In the spherical geometry, by Lemma 4.1 the relative position between $-\omega$ and the essential spectra $\sigma _{ess}(\mathcal {L}_{k,\omega })=Ran(-\Psi _{0}')$ affects the real spectra of the linearized operator $\mathcal {L}_{k,\omega }$, as shown in Fig. 8.

(ii) In Section 3, we highlighted differences between the rigidity of the travelling waves in the *f*-plane approximation (for $\beta =0$) and in the $\beta$-plane approximation (with $\beta >0$), the first case being much simpler: for $\beta =0$, the speed *c* of a zonally non-sheared travelling wave solution $\psi (x-ct,y)$ has to be in $Ran(-\psi _y)$ by Proposition 3.3. Since (2.13) yields

$$\begin{aligned} \beta =\frac{2L'\cos \theta _0}{R'}\,\omega \end{aligned}$$

as $\omega = \frac{\varOmega 'L'}{U'}$, we see that $\beta =0$ for a non-rotating sphere. However, for $\beta >0$, the propagation speed *c* can be slightly less than $(-\psi _y)_{\min }$, see Theorem 3.11 and Examples 3.12-3.13. In contrast to this, in the spherical geometry the case $\omega =0$ is not distinguished since only the relative position between $-\omega$ and the essential spectra $\sigma _{ess}(\mathcal {L}_{k,\omega })=Ran(-\Psi _{0}')$ of the linearized operator $\mathcal {L}_{k,\omega }$ around the background zonal flow $\Psi _0$ is important. Considering travelling wave solutions near a zonal flow $\Psi _0$ in the sense of (4.9), due to Theorem 4.2, we distinguish between three scenarios:

1. If $-\omega \in Ran(-\Psi _{0}')$, then the propagation speed *c* of an oscillating travelling wave solution $\Psi (\varphi -ct,s)$ near the zonal flow $\Psi _0$ has to be very close to $Ran(-\Psi _{0}')$.
2. If $-\omega >(-\Psi _{0}')_{\max }$, then the propagation speed *c* of an oscillating travelling wave solution $\Psi (\varphi -ct,s)$ can be slightly larger than $(-\Psi _{0}')_{\max }$. In this context, note that in [11] the existence of oscillating travelling wave solutions $\Psi (\varphi -ct,s)$ with speeds $c>(-\Psi _{0}')_{\max }$ near the 3-jet zonal flow is proved.
3. If $-\omega <(-\Psi _{0}')_{\min }$, then the propagation speed *c* of an oscillating travelling wave solution $\Psi (\varphi -ct,s)$ can be slightly less than $(-\Psi _{0}')_{\min }$. In particular, in [11] it is shown that there are oscillating travelling wave solutions $\Psi (\varphi -ct,s)$ with speeds $c<(-\Psi _{0}')_{\min }$ near the 3-jet zonal flow.

(iii) Another important difference is that our rigidity results in Theorems 3.2 and 3.11 for the flat-space geometry of the $\beta$-plane apply for travelling waves with arbitrarily large amplitude of the meridional velocity, not necessarily near a shear flow. However, our rigidity results in Theorem 4.2 for the spherical geometry only apply for travelling waves near a zonal flow, that is, they require the amplitude of the meridional velocity to be sufficiently small.

We now prove a rigidity which improves Theorem 3.12 in [10] if the minimal period of the travelling wave solution in the $\varphi$-direction is less than $2\pi$.

#### Proposition 4.4

Consider $\omega \ge 0$ and a travelling wave solution $\Psi (\varphi -ct,s)\in C^3(\mathbb {S}^2)$ of (4.1) satisfying (4.3) for some $G \in C^1(Ran(\Psi +cs))$. If the minimal period of $\Psi$ in the $\varphi$-direction is $\frac{2\pi }{k_0}$ for some integer $k_0 \ge 1$ and if $-G'<k_0(k_0+1)$, then $\Psi$ is a zonal flow.

#### Proof

Taking the derivative of (4.3) with respect to the $\varphi$-variable, we get

$$\begin{aligned} \Delta \Psi _\varphi =G'(\Psi +cs)\Psi _\varphi . \end{aligned}$$

Let

$$\begin{aligned}X=&\left\{ \Phi \bigg | \int _0^{{2\pi \over k_0}}\int _{-1}^{1}\left( (1-s^2)|\partial _s\Phi |^2+\frac{1}{1-s^2}|\partial _\varphi \Phi |^2\right) dsd\varphi<\infty \right\} ,\\ X_0=&\left\{ \Phi _0\bigg |\int _{-1}^{1}(1-s^2)|\Phi _0'(s)|^2ds<\infty \right\} ,\\ X_1=&\left\{ \Phi _1\bigg | \int _{-1}^{1}\left( (1-s^2)|\Phi _1'(s)|^2+\frac{1}{1-s^2}|\Phi _1(s)|^2\right) ds<\infty \right\} . \end{aligned}$$

We decompose the eigenvalue problem

$$\begin{aligned} \left\{ \begin{aligned}&-\Delta \Phi =\lambda \Phi ,\\&\Phi \in X, \end{aligned} \right. \end{aligned}$$

into the sequence of ordinary differential equations

4.29 $$\begin{aligned} \left\{ \begin{aligned}&-\left( (1-s^2)\Phi _0'\right) '=\lambda \Phi _0,\\&\Phi _0\in X_0, \end{aligned} \right. \end{aligned}$$

and

4.30 $$\begin{aligned} \left\{ \begin{aligned}&-\left( (1-s^2)\Phi _k'\right) '+\frac{(k_0k)^2}{1-s^2}\Phi _k=\lambda \Phi _k,\\&\Phi _k\in X_1, \end{aligned} \right. \end{aligned}$$

where $k\ne 0$. The sequence of the eigenvalues of (4.29) is $\{l(l+1)\}_{l\ge 0}$ and that of the eigenvalues of (4.30) for $k=1$ is $\{l(l+1)\}_{l\ge k_0}$. Thus, the principal eigenvalue of (4.30) for $k=1$ is $k_0(k_0+1)$ with a corresponding eigenfunction $\Phi _1^{(1)}$. The eigenvalues of (4.29) which are less than $k_0(k_0+1)$ are $\{l(l+1)\}_{0\le l<k_0}$ with corresponding eigenfunctions denoted by $\Phi _{1}^{(0)},\cdots ,\Phi _{k_0}^{(0)}$. Note that

$$\begin{aligned} \Psi _\varphi (\varphi ,s)=\sum _{k\ne 0}e^{ikk_0\varphi }\Psi _{\varphi ,k}(s). \end{aligned}$$

Denote $\Psi _1(\varphi ,s)=\Phi _1^{(1)}(s)e^{ik_0\varphi }$. Then by (4.30) for $k=1$ we have

4.31 $$\begin{aligned} -\partial _{s}\left( (1-s^2)\partial _{s}\Psi _1\right) -\frac{\partial _{\varphi }^2\Psi _1}{1-s^2}=k_0(k_0+1)\Psi _1. \end{aligned}$$

Integrating by parts and taking (4.31) into account, we obtain

4.32 $$\begin{aligned}&\left\Vert \sqrt{1-s^2}\partial _{s}\Phi -\Phi \frac{\sqrt{1-s^2}\partial _{s}\Psi _1}{\Psi _1} \right\Vert ^2_{L^2\left( (0,\frac{2\pi }{k_0})\times (-1,1)\right) }\\ \nonumber =&\int _{-1}^{1}\int _{0}^{\frac{2\pi }{k_0}}\left( (1-s^2)|\partial _{s}\Phi |^2 -2(1-s^2)\Phi \partial _{s}\Phi \frac{\partial _{s}\Psi _1}{\Psi _1} +\Phi ^2\frac{(1-s^2)|\partial _{s}\Psi _1|^2}{|\Psi _1|^2}\right) d\varphi ds\\ \nonumber =&\int _{-1}^{1}\int _{0}^{\frac{2\pi }{k_0}} \left( (1-s^2)|\partial _{s}\Phi |^2-(1-s^2)\partial _{s}\Psi _1\partial _{s}\left( \frac{\Phi ^2}{\Psi _1}\right) \right) d\varphi ds\\ \nonumber =&\int _{-1}^{1}\int _{0}^{\frac{2\pi }{k_0}}(1-s^2)|\partial _{s}\Phi |^2d\varphi ds -\int _{0}^{\frac{2\pi }{k_0}}\bigg ((1-s^2)\frac{\Phi ^2}{\Psi _1}\partial _{s}\Psi _1\bigg |_{s=-1}^{1}\\ \nonumber&-\int _{-1}^{1}\frac{\partial _{s}\left( (1-s^2)\partial _{s}\Psi _1\right) \Phi ^2}{\Psi _1}ds\bigg )d\varphi \\ \nonumber =&\int _{-1}^{1}\int _{0}^{\frac{2\pi }{k_0}}\left( (1-s^2)|\partial _{s}\Phi |^2 -\frac{\partial _{\varphi }^{2}\Psi _1\Phi ^2}{(1-s^2)\Psi _1}\right) d\varphi ds -\int _{-1}^{1}\int _{0}^{\frac{2\pi }{k_0}}k_0(k_0+1)\Phi ^2d\varphi ds\\ \nonumber =&\int _{-1}^{1}\int _{0}^{\frac{2\pi }{k_0}}\left( (1-s^2)|\partial _{s}\Phi |^2+\frac{k_0^2|\Phi |^2}{1-s^2}\right) d\varphi ds-\int _{-1}^{1}\int _{0}^{\frac{2\pi }{k_0}}k_0(k_0+1)\Phi ^2d\varphi ds\ge 0 \end{aligned}$$

for any $\Phi (\varphi ,s)=\sum \limits _{k\ne 0}e^{ikk_0\varphi }\Phi _{k}(s) \in X$. By (4.31) and (4.32), we have

$$\begin{aligned} k_0(k_0+1)=\inf _{\Phi \in X,\;\Phi =\sum _{k\ne 0}e^{ikk_0\varphi }\Phi _{k}(s)} \frac{\int _{-1}^{1}\int _{0}^{\frac{2\pi }{k_0}}\left( (1-s^2)|\partial _{s}\Phi |^2+\frac{k_0^2|\Phi |^2}{1-s^2}\right) d\varphi ds}{\int _{-1}^{1}\int _{0}^{\frac{2\pi }{k_0}}|\Phi |^2d\varphi ds}, \end{aligned}$$

which yields

$$\begin{aligned} \frac{\int _{-1}^{1}\int _{0}^{\frac{2\pi }{k_0}}\left( (1-s^2)|\Psi _{\varphi s}|^2+\frac{k_0^2|\Psi _{\varphi }|^2}{1-s^2}\right) d\varphi ds}{\int _{-1}^{1}\int _{0}^{\frac{2\pi }{k_0}}|\Psi _\varphi |^2d\varphi ds} \ge k_0(k_0+1). \end{aligned}$$

Thus,

4.33 $$\begin{aligned}&\int _{-1}^{1}\int _{0}^{\frac{2\pi }{k_0}}\left( (1-s^2)|\Psi _{\varphi s}|^2+\frac{|\Psi _{\varphi \varphi }|^2}{1-s^2}\right) d\varphi ds\\\nonumber \ge&\int _{-1}^{1}\int _{0}^{\frac{2\pi }{k_0}}\left( (1-s^2)|\Psi _{\varphi s}|^2+\frac{k_0^2|\Psi _{\varphi }|^2}{1-s^2}\right) d\varphi ds \ge k_0(k_0+1)\int _{-1}^{1}\int _{0}^{\frac{2\pi }{k_0}}|\Psi _\varphi |^2d\varphi ds. \end{aligned}$$

If $$\Psi _\varphi \not \equiv 0$$, then

$$\begin{aligned}&\int _{-1}^{1}\int _{0}^{\frac{2\pi }{k_0}}\left( (1-s^2)|\Psi _{\varphi s}|^2+\frac{|\Psi _{\varphi \varphi }|^2}{1-s^2}\right) d\varphi ds =\int _{-1}^{1}\int _{0}^{\frac{2\pi }{k_0}}|\nabla \Psi _\varphi |^2d\varphi ds\\ =&\int _{-1}^{1}\int _{0}^{\frac{2\pi }{k_0}}-\Delta \Psi _\varphi \Psi _\varphi d\varphi ds =\int _{-1}^{1}\int _{0}^{\frac{2\pi }{k_0}}-G'(\Psi +cs)|\Psi _\varphi |^2d\varphi ds\\ <&k_0(k_0+1)\int _{-1}^{1}\int _{0}^{\frac{2\pi }{k_0}}|\Psi _\varphi |^2d\varphi ds, \end{aligned}$$

which contradicts (4.33). Hence, $\Psi _\varphi \equiv 0$ and $\Psi (\varphi -ct,s)$ is a zonal flow. $\square$

#### Remark 4.5

Proposition 4.4 is optimal in the sense that for any $\omega >0$, any $c\in \mathbb {R}$ and any $k_0\ge 1$, there exist genuine travelling wave solutions $\Psi (\varphi -ct,s)$ satisfying (4.3) with $-G'=k_0(k_0+1)$ and minimal period ${2\pi \over k_0}$ in the $\varphi$-direction. Indeed, note that $k_0(k_0+1)$ is an eigenvalue of $-\Delta$ with a corresponding eigenfunction $\cos (k_0\varphi )P_{k_0}^{k_0}(s)$. With

$$\begin{aligned} -c={2\omega \over {k_0(k_0+1)}}+{\alpha (k_0(k_0+1)-2)\over k_0(k_0+1)}, \end{aligned}$$

we see that $\Psi (\varphi -ct,s)=\alpha s+\cos (k_0(\varphi -ct))P_{k_0}^{k_0}(s)$ is a non-sheared travelling wave solution.

The function *G* in (4.3) is not necessarily invertible. However, the stream function can be written as a function of vorticity if $2\omega$ is not in the range of $-\partial _s\Upsilon$.

#### Lemma 4.6

For a travelling wave solution $\Psi (\varphi -ct,s)\in C^3(\mathbb {S}^2)$ of (4.2) with $2\omega \notin Ran(-\partial _s\Upsilon )$, there exists $F\in C^1(Ran(\Upsilon +2\omega s))$ such that

4.34 $$\begin{aligned} \Psi +cs=F(\Upsilon +2\omega s)=F(\Delta \Psi +2\omega s), \end{aligned}$$

where $$\partial _s\Psi , \partial _s\Upsilon \in C(\mathbb {S}^2)$$.

#### Proof

We change the variables $(\varphi ,s)$ to be

$$\begin{aligned} \hat{q}=\varphi \quad \text {and}\quad p=\Upsilon (\varphi ,s)+2\omega s. \end{aligned}$$

Then $${\partial {(\hat{q},p)}\over \partial {(\varphi ,s)}}=\partial _s\Upsilon +2\omega \ne 0$$,

$$\begin{aligned} \partial _\varphi =\partial _{\hat{q}}+\partial _\varphi \Upsilon \partial _p\quad \text {and}\quad \partial _s=(\partial _s\Upsilon +2\omega )\partial _p. \end{aligned}$$

Thus, $\partial _{\hat{q}}=\partial _\varphi -\partial _\varphi \Upsilon {\partial _s\over \partial _s\Upsilon +2\omega }$ and $\partial _p={\partial _s \over \partial _s\Upsilon +2\omega }$. By (4.2), $\Psi$ solves

$$\begin{aligned} \partial _{\varphi }(\Psi +cs)(\partial _{s}\Upsilon +2\omega )-\partial _{s}(cs+\Psi )\partial _{\varphi }\Upsilon =0. \end{aligned}$$

Then

$$\begin{aligned} \partial _{\hat{q}}(\Psi +cs)=&\left( \partial _\varphi -\partial _\varphi \Upsilon {\partial _s\over \partial _s\Upsilon + 2\omega }\right) (\Psi +cs)\\ =&{\partial _{\varphi }(\Psi +cs)(\partial _{s}\Upsilon +2\omega )-\partial _{s}(cs+\Psi )\partial _{\varphi }\Upsilon \over 2\omega +\partial _s\Upsilon }=0. \end{aligned}$$

This, together with $\Upsilon \in C^1$ and $\Psi \in C^3$, implies that there exists a function $F\in C^1$ such that $\Psi +cs=F(p)=F(\Upsilon +2\omega s)$ throughout the fluid. $\square$

Then we provide a rigidity result that relies on (4.34).

#### Proposition 4.7

Let $\omega \ge 0$ and let $\Psi (\varphi -ct,s)\in C^3(\mathbb {S}^2)$ be a travelling wave solution of (4.2) satisfying (4.34) for some $F\in C^1(Ran(\Upsilon +2\omega s))$, where $\partial _s\Upsilon \in C(\mathbb {S}^2)$ and $\partial _\varphi \Psi \in H_1^2(\mathbb {S}^2)$. If the minimal period of $\Psi$ in the $\varphi$-direction is $\frac{2\pi }{k_0}$ for some integer $k_0 \ge 1$, and *F* satisfies $F'\ge 0$ or $F'<-1/(k_0(k_0+1))$, then $\Psi (\varphi -ct,s)$ is a zonal flow.

#### Proof

Since *G* is not necessarily invertible in Proposition 4.4, this proposition is not its direct consequence.

Taking the derivative of (4.34) with respect to the $\varphi$-variable, we obtain

4.35 $$\begin{aligned} \Psi _\varphi =F'(\Delta \Psi +2\omega s)\Delta \Psi _\varphi . \end{aligned}$$

Note that the minimal period of $\Psi _\varphi$ in the $\varphi$-direction is $\frac{2\pi }{k_0}$ and its zero’th Fourier mode is 0. Thus, we can apply (4.33) to obtain

$$\begin{aligned}&\int _{-1}^{1}\int _{0}^{\frac{2\pi }{k_0}}|\nabla \Psi _{\varphi }|^2d\varphi ds=-\int _{-1}^{1}\int _{0}^{\frac{2\pi }{k_0}}\Psi _\varphi \Delta \Psi _{\varphi }d\varphi ds\\ \le&\left( \int _{-1}^{1}\int _{0}^{\frac{2\pi }{k_0}}|\Psi _\varphi |^2 d \varphi ds\right) ^{1\over 2}\left( \int _{-1}^{1}\int _{0}^{\frac{2\pi }{k_0}}|\Delta \Psi _{\varphi }|^2 d\varphi ds\right) ^{1\over 2}\\ \le&{1\over \sqrt{k_0(k_0+1)}}\left( \int _{-1}^{1}\int _{0}^{\frac{2\pi }{k_0}}\left( (1-s^2)|\Psi _{\varphi s}|^2+\frac{|\Psi _{\varphi \varphi }|^2}{1-s^2}\right) d\varphi ds\right) ^{1\over 2}\left( \int _{-1}^{1}\int _{0}^{\frac{2\pi }{k_0}}|\Delta \Psi _{\varphi }|^2 d\varphi ds\right) ^{1\over 2}\\ =&{1\over \sqrt{k_0(k_0+1)}}\left( \int _{-1}^{1}\int _{0}^{\frac{2\pi }{k_0}}|\nabla \Psi _{\varphi }|^2d\varphi ds\right) ^{1\over 2}\left( \int _{-1}^{1}\int _{0}^{\frac{2\pi }{k_0}}|\Delta \Psi _{\varphi }|^2 d\varphi ds\right) ^{1\over 2}. \end{aligned}$$

Then

$$\begin{aligned} \int _{-1}^{1}\int _{0}^{\frac{2\pi }{k_0}}|\nabla \Psi _{\varphi }|^2d\varphi ds \le&{1\over k_0(k_0+1)}\int _{-1}^{1}\int _{0}^{\frac{2\pi }{k_0}}|\Delta \Psi _{\varphi }|^2 d\varphi ds. \end{aligned}$$

By (4.35), we have

4.36 $$\begin{aligned} -\int _{-1}^{1}\int _{0}^{\frac{2\pi }{k_0}}|\nabla \Psi _{\varphi }|^2d\varphi ds=\int _{-1}^{1}\int _{0}^{\frac{2\pi }{k_0}}F'(\Delta \Psi +2\omega s)|\Delta \Psi _\varphi |^2d\varphi ds. \end{aligned}$$

If $F'\ge 0$, then it follows from (4.36) and $\Psi _\varphi (\cdot ,\pm 1)=0$ that $\Psi _\varphi =0$ on $\mathbb {T}_{{2\pi \over k_0}}\times [-1,1]$. Thus, $\Psi (\varphi -ct,s)$ is a zonal flow.

For the case $F'< -{1\over k_0(k_0+1)}$, if $\Delta \Psi _{\varphi }\not \equiv 0$, then

$$\begin{aligned}&-{1\over k_0(k_0+1)}\int _{-1}^{1}\int _{0}^{\frac{2\pi }{k_0}}|\Delta \Psi _{\varphi }|^2 d\varphi ds \le -\int _{-1}^{1}\int _{0}^{\frac{2\pi }{k_0}}|\nabla \Psi _{\varphi }|^2d\varphi ds\\ =&\int _{-1}^{1}\int _{0}^{\frac{2\pi }{k_0}}F'(\Delta \Psi +2\omega s)|\Delta \Psi _\varphi |^2d\varphi ds<-{1\over k_0(k_0+1)}\int _{-1}^{1}\int _{0}^{\frac{2\pi }{k_0}}|\Delta \Psi _{\varphi }|^2 d\varphi ds. \end{aligned}$$

Thus, $\Delta \Psi _\varphi =0$, $\Psi _\varphi =0$ and $\Psi (\varphi -ct,s)$ is a zonal flow. $\square$

### Asymmetric travelling wave solutions in spherical coordinates

In this subsection, we construct examples of steady solutions to (4.1) that are not symmetric, up to any translation, in the $\varphi$-direction. Due to the richness of spherical harmonic functions, this construction is simpler than that of the plane in Subsection 3.11.2. We only construct asymmetric steady flows. Travelling wave solutions can be constructed similarly.

#### Example 4.8

Consider the eigenvalue problem $\Delta \psi =-j(j+1)\psi$ for the Laplace-Beltrami operator. For $j=5$ we choose the spherical harmonic functions

$$\begin{aligned}R_{5}^{5}(\varphi ,\theta )=\cos ^{5}(\theta )\cos (5\varphi ),\quad R_{5}^{1}(\varphi ,\theta )=(21\sin ^{4}(\theta )-14\sin ^{2}(\theta )+1)\cos (\theta )\sin (\varphi ).\end{aligned}$$

Let $$\alpha =-\omega /14$$ and

$$\begin{aligned}Y(\varphi ,\theta )=R_{5}^{5}(\varphi ,\theta )+R_{5}^{1}(\varphi ,\theta ),\qquad \psi (\varphi ,\theta )=\alpha \sin \theta +Y(\varphi ,\theta ).\end{aligned}$$

**Fig. 9:**
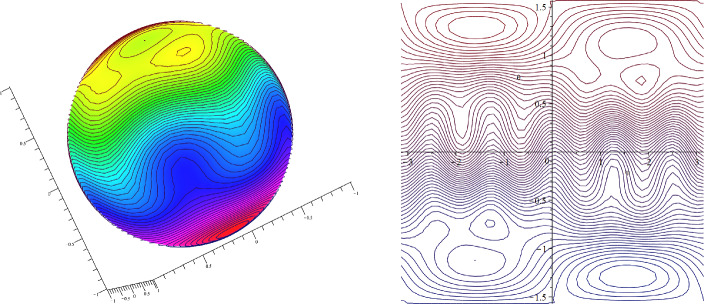
Zonally asymmetric steady flow in spherical coordinates

Then $\psi (\varphi ,\theta )$ is a Rossby-Haurwitz steady solution of (4.1). For $\omega =98$ and

$$\begin{aligned}\psi (\varphi ,\theta )=-7\sin (\theta )+\cos ^{5}(\theta )\cos (5\varphi )+(21\sin ^{4}(\theta )-14\sin ^{2}(\theta )+1)\cos (\theta )\sin (\varphi ),\end{aligned}$$

the streamline pattern on the sphere and on the $( \varphi ,\theta )$-plane, depicted in Fig. 9, of this steady flow is not symmetric, up to any translation, in the $\varphi$-direction.

#### Example 4.9

**Symmetry-breaking in a global sense due to the rotation rate.**

Note that

**Fig. 10:**
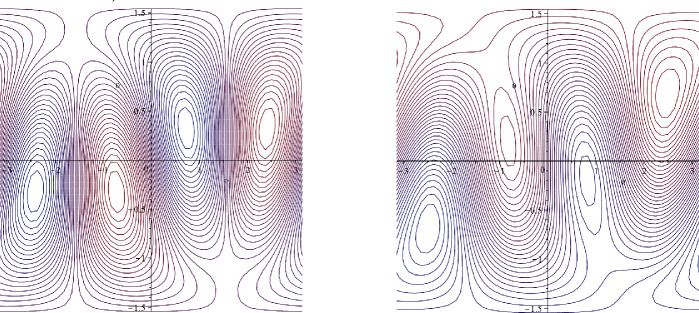
Streamlines of the steady solution (4.37) for $\omega =0$ (left) and for $\omega =2$ (right). The level set $\{\psi =0\}$ is symmetric in the $\varphi$-direction for $\omega =0$, but the symmetry is globally broken for $\omega =2.$

4.37 $$\begin{aligned} \psi (\varphi ,\theta )=-{\omega \over 2}\sin \theta +Y(\varphi ,\theta )\end{aligned}$$

is a steady solution of (4.1), where $Y(\varphi ,\theta )=R_{2}^{1}(\varphi ,\theta )+R_{2}^{2}(\varphi ,\theta )$, $R_{2}^{1}(\varphi ,\theta )=\sin (\theta )\cos (\theta )$ $\cos (\varphi )$ and $R_{2}^{2}(\varphi ,\theta )=\cos ^{2}(\theta )\sin (2\varphi )$. In Fig. 10, the streamline pattern shows the global symmetry-breaking due to the rotation effect.

## Data Availability

All data for this paper are properly cited and referred to: the relevant data can be found in [2, 21, 26, 32, 37].
